# Symptom clusters and symptom network characteristics in chronic fatigue syndrome patients: a cross-sectional study

**DOI:** 10.3389/fmed.2026.1955021

**Published:** 2026-10-20

**Authors:** Aiqun Zi, Yihan Zhang, Min Sun, Ting Liu, Yan Xu, Min Xu

**Affiliations:** 1 School of Nursing, Zhejiang Chinese Medical University, Hangzhou, Zhejiang, China; 2 Department of Nursing, The First Affiliated Hospital, Zhejiang Chinese Medical University, Hangzhou, Zhejiang, China

**Keywords:** chronic fatigue syndrome, core symptoms, symptom burden, symptom cluster, symptom network

## Abstract

**Background:**

Chronic fatigue syndrome is characterized by multiple co-occurring physical, cognitive, sleep, and emotional symptoms. Traditional symptom cluster analysis identifies patterns of symptom co-occurrence but does not clarify the relative importance of individual symptoms or the connections between symptom clusters. Symptom network analysis may complement this approach by identifying core and bridge symptoms.

**Objective:**

To investigate the symptom clusters and structural characteristics of symptom networks in patients with chronic fatigue syndrome, thereby providing a basis for developing targeted strategies for managing symptoms.

**Methods:**

A convenience sampling method was used to enroll 214 patients with chronic fatigue syndrome. Data were collected using a demographic questionnaire and an adapted version of the Chinese Memorial Symptom Assessment Scale. Data were analyzed using SPSS 27.0. Symptom clusters were extracted using principal component analysis (PCA) with varimax rotation, and a symptom network was constructed in R to calculate expected influence and bridge expected influence, thereby identifying core and bridge symptoms.

**Results:**

The three most common symptoms, in descending order, were fatigue (100.00%), difficulty concentrating (90.7%), and feeling drowsy (86.9%). A total of six symptom clusters were identified: Emotional-Sleep Symptom cluster, Fatigue Symptom cluster, Physical symptom cluster, Self-Image symptom cluster, Gastrointestinal symptom cluster, and Neurological symptom cluster, with a cumulative variance contribution of 63.114%. Fatigue had the highest expected influence (rEI = 1.541), followed by worrying (rEI = 1.169); fatigue (rbEI = 0.430), pain (rbEI = 0.363), and difficulty sleeping (rbEI = 0.309) were the primary bridge symptoms.

**Conclusion:**

Patients with chronic fatigue syndrome exhibit co-occurring symptoms and connections across symptom clusters. Fatigue serves as both a core symptom and a bridging symptom, while worrying, difficulty sleeping, and pain are also critical areas of focus in symptom management. Clinically, fatigue should be prioritized for assessment and management, while concurrently conducting comprehensive evaluations of mood, sleep, and pain. Individualized management plans should be developed based on the characteristics of symptom clusters and symptom networks to alleviate the symptom burden in patients with chronic fatigue syndrome and improve their quality of life.

## 1 Introduction

Chronic Fatigue Syndrome (CFS) is also known as Myalgic Encephalomyelitis (ME). The 1994 Fukuda case definition describes CFS as unexplained fatigue lasting at least 6 months, accompanied by at least four of eight symptoms: impaired memory or concentration, sore throat, tender cervical or axillary lymph nodes, muscle pain, multijoint pain without swelling or redness, headaches of a new type, pattern, or severity, non-restorative sleep, and post-exertional malaise lasting more than 24 h (1). Studies have found that the prevalence of CFS is approximately 0.2%–2.2% (2), with an estimated 17–24 million people worldwide diagnosed with the condition (3). CFS is more frequently reported among individuals aged 30–49, and the prevalence among women is approximately 1.5–2 times that of men (4). CFS can significantly impair patients' health-related quality of life (5) and impose a heavy family and socioeconomic burden (6). The condition typically manifests as a coexistence of multiple physical, cognitive, sleep, and emotional symptoms; assessing individual symptoms alone cannot fully reflect patients' symptom experiences. A symptom cluster refers to two or more interrelated symptoms that occur simultaneously (7); identifying symptom clusters helps understand the patterns of symptom co-occurrence from a holistic perspective. Symptom network analysis visualizes conditional associations among symptoms, enabling the identification of core symptoms with significant influence within the network as well as bridge symptoms that connect different symptom clusters (8). This study employed principal component analysis to identify symptom clusters in CFS patients and further constructed a symptom network, with the aim of providing a basis for developing stratified and precision symptom management strategies for clinical practice.

## 2 Methods

### 2.1 Study design

We conducted this cross-sectional study from September 18, 2025 to April 2026. We collected data from 214 patients with CFS at the Traditional Chinese Medicine Internal Medicine Outpatient Clinic of The First Affiliated Hospital of Zhejiang Chinese Medical University in Zhejiang Province, China. The study was approved by the Ethics Review Committee of the First Affiliated Hospital of Zhejiang Chinese Medical University (approval number: 2025-KLS-637-01; approval date: September 17, 2025), before participant recruitment commenced.

### 2.2 Study population

Inclusion Criteria: ① Meets the 1994 U.S. Centers for Disease Control and Prevention (CDC) diagnostic criteria for chronic fatigue syndrome (1); ② Age≥18 years; ③ Possesses normal comprehension and expression abilities and can complete scales and questionnaires independently or with the assistance of a researcher; ④ Has provided informed consent and voluntarily participates in this study. Exclusion Criteria: ① Having serious and uncontrolled health issues related to the heart, lungs, kidneys, digestive system, blood, mental health, immune system, or metabolism, as well as cancers, severe malnutrition, or other conditions that could impact symptom evaluation or survival; ② Pregnant or breastfeeding women; ③ Individuals whose mental state prevents them from cooperating with the assessment, who lack insight, or who are unable to express themselves clearly.

### 2.3 Sample size

For factor analysis, the recommended sample size is typically 5–10 times the number of items, with a minimum of 100 cases. The revised Memorial Symptom Assessment Scale used in this study contains 35 symptom items; based on these items, the estimated sample size was 175–350 participants. Taking into account a 10% non-response rate, the planned sample size was set at 195–389 participants. A total of 225 questionnaires were distributed, and 214 valid responses were ultimately obtained.

### 2.4 Measures

#### 2.4.1 General information questionnaire

A self-designed general information questionnaire was used to collect data on age, sex, body mass index, educational level, marital status, monthly income, smoking history, alcohol consumption history, physical activity status, and physical activity intensity.

#### 2.4.2 Memorial symptom assessment scale (MSAS)

The revised Memorial Symptom Assessment Scale (MSAS) includes a total of 35 common symptoms. The literature findings and patient feedback were reviewed by the research team to determine the final modifications. Four new symptoms: Bradyphrenia, Chills, Noise sensitivity, and palpitations were added to the original 32 symptoms. Preliminary survey results indicated that most patients avoided or refused to answer the item “Problems with sexual interest or activity” on the revised MSAS due to privacy concerns; after comprehensive consideration by the research team, it was decided to remove this item from the formal survey. The adaptation process and item-specific rationale are provided in Supplementary Table S1. In the revised MSAS, 27 symptoms are assessed based on frequency, severity, and distress; the remaining 8 symptoms are assessed only for severity and distress. Each symptom is recorded as present or absent; if present, a four- to five-point rating scale is used to assess the symptom's frequency, severity, and associated distress over the past 7 days. A higher score indicates greater frequency, more severe intensity, and more pronounced distress associated with the symptom. In this study, the Cronbach's *α* coefficient for the MSAS was 0.949, with Cronbach's *α* values for the dimensions of frequency, severity, and distress at 0.822, 0.839, and 0.807, respectively, indicating that the scale possesses excellent internal consistency.

### 2.5 Data collection and quality control

Two trained researchers conducted the survey. Before questionnaire administration, they explained the study purpose, procedures, expected time commitment, and the nature of questionnaire data collection to the patients. Patients were informed that participation was voluntary, that they could decline to participate or withdraw at any time without affecting their medical care, and that their information would be kept confidential and used only for research purposes. Patients were given an opportunity to ask questions, which were answered by the researchers before enrollment. Written informed consent was obtained from each participant before data collection. For patients who were unable to write due to various reasons, the researchers completed the questionnaire on their behalf based solely on the patients' verbal responses, without interpreting or suggesting answers. Completed questionnaires were collected on the spot, and their completeness was verified immediately. A total of 225 questionnaires were distributed; 11 invalid questionnaires—characterized by repetitive responses or completely identical selections—were excluded, resulting in 214 valid questionnaires and a valid response rate of 95.11%.

### 2.6 Data analysis

Data were analyzed using SPSS 27.0. Data following a normal distribution were reported as mean ± standard deviation, while data from skewed distributions were reported as medians and interquartile ranges. The factor structure of the adapted MSAS symptom items was examined using exploratory factor analysis. Items with no variance were excluded. Principal-axis factoring with Promax rotation was applied, and the number of factors was determined using permutation-based parallel analysis. The detailed results of this exploratory factor analysis are presented in Supplementary Tables S2.1–S2.3. Separately, symptoms with a prevalence of ≥35% were included in the principal component analysis. The criteria for identifying symptom clusters were: an eigenvalue ≥1, at least two symptoms per component, and component loadings ≥0.40; if a symptom had loadings ≥0.40 on multiple components, it was assigned to the component with the highest loading. Varimax rotation was performed to facilitate interpretation of the components.

Symptom network analysis was conducted using R version 4.5.3. A Gaussian graphical model (GGM) was estimated using the EBICglasso method with an EBIC tuning parameter of 0.50, with correlations calculated by the cor_auto function. The qgraph package was used for network visualization. Nodes in the network represent symptoms, and edges represent regularized partial correlations after controlling for other symptoms; thicker edges indicate stronger conditional associations. Expected influence (EI) and bridge expected influence (bridge EI) were calculated. The six symptom clusters identified by PCA were used as communities for calculating bridge expected influence using the bridge function in the networktools package. This approach was used to examine connections across the PCA-derived symptom clusters. The node-to-community assignments are presented in Supplementary Table S8a. Higher EI values indicate greater net connectivity between a symptom and the overall network, whereas higher bridge EI values indicate greater net connectivity between a symptom and symptoms in other communities.

The bootnet package was used to perform 1,000 nonparametric bootstrap resamples to estimate the 95% confidence intervals for edge weights, and the correlation stability coefficient (CS) was calculated using case-dropping bootstrapping. A CS >0.25 is considered acceptable, while a CS >0.50 indicates good stability (9). The significance level was set at *α* = 0.05.

Potential node redundancy was assessed across all 231 symptom pairs using the Goldbricker procedure in the networktools package, based on correlations calculated by cor_auto. Using the Hittner method at *p* < 0.05, pairs were flagged when their correlation exceeded 0.50 and fewer than 25% of their correlations with the remaining symptoms differed significantly. Screening results were considered alongside item content to determine node retention. Sensitivity analyses were conducted by separately removing each distinct node involved in a flagged pair. Feeling drowsy was additionally removed in a separate model to examine its potential overlap with fatigue. Each reduced network was re-estimated using the same EBICglasso specification, while retaining the community assignments of the remaining nodes. EI and bridge EI rankings among common nodes were compared with those in the primary network using Spearman correlations. Detailed screening results and sensitivity comparisons are presented in Supplementary Tables S8b,c.

As an exploratory sensitivity analysis, each symptom severity score was regressed on age and sex. Age was entered as a continuous covariate, and sex was entered as a categorical covariate. A Pearson correlation network was then estimated from the residuals using EBICglasso with the same tuning parameter. An unadjusted Pearson correlation network based on the same 214 participants was used as the reference network. The same 22 symptom nodes and community assignments were retained across the three networks.

## 3 Results

### 3.1 Participant characteristics

Of the 214 patients, 142 were female (66.36%); the majority were aged 31–40 (43.46%). See Table 1 for details.

**Table 1:** Participant characteristics (*N* = 214).

Variable	*n* (%)	Variable	*n* (%)
Sex		BMI	
Male	72 (33.64)	Underweight	28 (13.08)
Female	142 (66.36)	Normal weight	158 (73.83)
Age (years)		Overweight	20 (9.35)
18–30	78 (36.45)	Obesity	8 (3.74)
31–40	93 (43.46)	Smoking history	
41–50	28 (13.08)	Never smoked	169 (78.97)
51–60	15 (7.01)	Current smoker	36 (16.82)
Educational level		Former smoker	9 (4.21)
Primary school or below	2 (0.93)	Alcohol consumption history	
Junior high school	16 (7.48)	Never drank alcohol	146 (68.22)
Senior high school/technical secondary school	13 (6.07)	Occasional alcohol consumption	65 (30.37)
College/undergraduate	146 (68.22)	Frequent alcohol consumption	3 (1.40)
Master's degree or above	37 (17.29)	Physical activity status	
Marital status		Predominantly sedentary (<5,000 steps/day)	97 (45.33)
Divorced/widowed	2 (0.93)	Light activity (5,000–7,499 steps/day)	72 (33.64)
Unmarried	69 (32.24)	Moderate activity (7,500–9,999 steps/day)	29 (13.55)
Married/cohabiting	143 (66.82)	High activity (≥10,000 steps/day)	16 (7.48)
Monthly income (CNY)		Physical activity intensity	
<3,000	26 (12.15)	Light-intensity physical activity	172 (80.37)
3,000–6,000	24 (11.21)	Moderate-intensity physical activity	38 (17.76)
6,000–9,000	92 (42.99)	High-intensity physical activity	4 (1.87)
9,000–12,000	30 (14.02)		
>12,000	42 (19.63)		

### 3.2 Symptom prevalence, frequency, severity, and distress

The survey results indicate that the top three symptoms among CFS patients, in order, are Fatigue (100.00%), Difficulty concentrating (90.7%), and Feeling drowsy (86.9%). The prevalence and severity of the symptoms are presented in Table 2. Detailed data on the prevalence, frequency, severity, and distress of all symptoms are provided in Supplementary Table S3.

**Table 2:** Symptom prevalence, severity (*N* = 214).

Symptom	Prevalence (*n*, %)	Severity
		Mean (SD)
Difficulty concentrating	194 (90.7)	1.77 (0.81)
Pain	159 (74.3)	1.74 (0.80)
Fatigue	214 (100)	1.94 (0.93)
Cough	72 (33.6)	1.35 (0.61)
Feeling nervous	155 (72.4)	2.28 (0.99)
Dry mouth	148 (69.2)	1.53 (0.75)
Nausea	55 (25.7)	1.29 (0.57)
Feeling drowsy	186 (86.9)	2.11 (0.79)
Numbness/tingling in hands/feet	67 (31.3)	1.15 (0.36)
Difficulty sleeping	129 (60.3)	1.59 (0.74)
Feeling bloated	123 (57.5)	1.63 (0.65)
Problems with urination	5 (2.3)	2.20 (0.84)
Vomiting	13 (6.1)	1.31 (0.48)
Shortness of breath	99 (46.3)	2.31 (1.00)
Diarrhea	73 (34.1)	1.58 (0.66)
Feeling sad	158 (73.8)	1.88 (0.72)
Sweating	124 (57.9)	1.83 (0.88)
Worrying	158 (73.8)	1.72 (0.78)
Itching	65 (30.4)	1.62 (0.63)
Lack of appetite	112 (52.3)	1.70 (0.77)
Dizziness	113 (52.8)	1.64 (0.72)
Difficulty swallowing	0 (0.0)	0.00 (0.00)
Feeling irritable	144 (67.3)	1.60 (0.61)
Palpitations	95 (44.4)	1.84 (0.96)
Bradyphrenia	150 (70.1)	1.77 (0.81)
Chills	118 (55.1)	1.53 (0.57)
Noise sensitivity	148 (69.2)	1.67 (0.68)
Mouth sores	63 (29.4)	1.43 (0.67)
Change in the way food tastes	61 (28.5)	1.36 (0.58)
Weight loss	26 (12.1)	1.42 (0.81)
Hair loss	93 (43.5)	1.65 (0.73)
Constipation	109 (50.9)	1.64 (0.73)
Swelling of arms or legs	26 (12.1)	1.23 (0.43)
“I don’t look like myself”	105 (49.1)	1.59 (0.76)
Skin changes	131 (61.2)	1.72 (0.81)

### 3.3 Symptom clusters based on symptom scores

Principal component analysis was performed on the 23 included symptoms. The results indicated that the factor loading for “Dry mouth” was below 0.4. After excluding this item, the maximum variance method was reapplied for rotation, resulting in a Kaiser–Meyer–Olkin (KMO) value of 0.787 and a Bartlett's sphericity test *χ*^2^ = 1,625.864, *P* < 0.001, indicating that the data were suitable for factor analysis. Using principal component extraction followed by varimax rotation, a total of six factors with eigenvalues >1 were extracted. These were named the “Emotional-Sleep Symptom Cluster,” “Fatigue Symptom Cluster,” “Physical Symptom Cluster,” “Self-Image Symptom Cluster,” “Gastrointestinal Symptom Cluster,” and “Neurological Symptom Cluster,” with a cumulative variance explained of 63.114%; see Table 3 for details. The complete factor loading matrix is provided in Supplementary Table S4.

**Table 3:** Symptom clusters based on symptom scores.

Symptom clusters	Symptom	Factor load	Cronbach's *α*
Emotional-sleep symptom cluster	A1 Feeling nervous	0.819	0.829
	A2 Feeling irritable	0.564	
	A3 Feeling sad	0.853	
	A4 Difficulty sleeping	0.578	
	A5 Worrying	0.836	
Fatigue symptom cluster	B1 Difficulty concentrating	0.743	0.792
	B2 Bradyphrenia	0.681	
	B3 Fatigue	0.810	
	B4 Feeling drowsy	0.678	
	B5 Pain	0.620	
Physical symptom cluster	C1 Shortness of breath	0.692	0.704
	C2 Chills	0.752	
	C3 Palpitations	0.736	
	C4 Sweating	0.635	
Self-image symptom cluster	D1 Skin changes	0.765	0.756
	D2 “I don’t look like myself”	0.835	
	D3 Hair loss	0.825	
Gastrointestinal symptom cluster	E1 Constipation	0.857	0.738
	E2 Feeling bloated	0.653	
	E3 Lack of appetite	0.862	
Neurological symptom cluster	F1 Dizziness	0.819	0.677
	F2 Noise sensitivity	0.832	

### 3.4 Symptom networks and centrality indicators

A network analysis was conducted on 22 symptom items representative of the symptom experiences of CFS patients. As indicated by the edge weights of the symptom network, the strongest estimated edges were observed between “Constipation” and “Lack of appetite”; between “I don’t look like myself” and “Hair loss”; between “Shortness of breath” and “Palpitations”. The symptom network structure is shown in Figure 1. The results of the centrality indices for symptom nodes indicate that Fatigue has the highest expected influence (rEI = 1.541), followed by Worrying (rEI = 1.169) and Lack of appetite (rEI = 1.072), indicating relatively high estimated centrality within this network. See Figure 2. The largest expected influence for bridges was Fatigue (rbEI = 0.430), followed by Pain (rbEI = 0.363) and Difficulty sleeping (rbEI = 0.309), indicating that these symptoms are the bridge symptoms in the symptom cluster network. See Figure 3. Full centrality indices for all 22 symptom nodes are shown in Supplementary Table S5.

**Figure 1:**
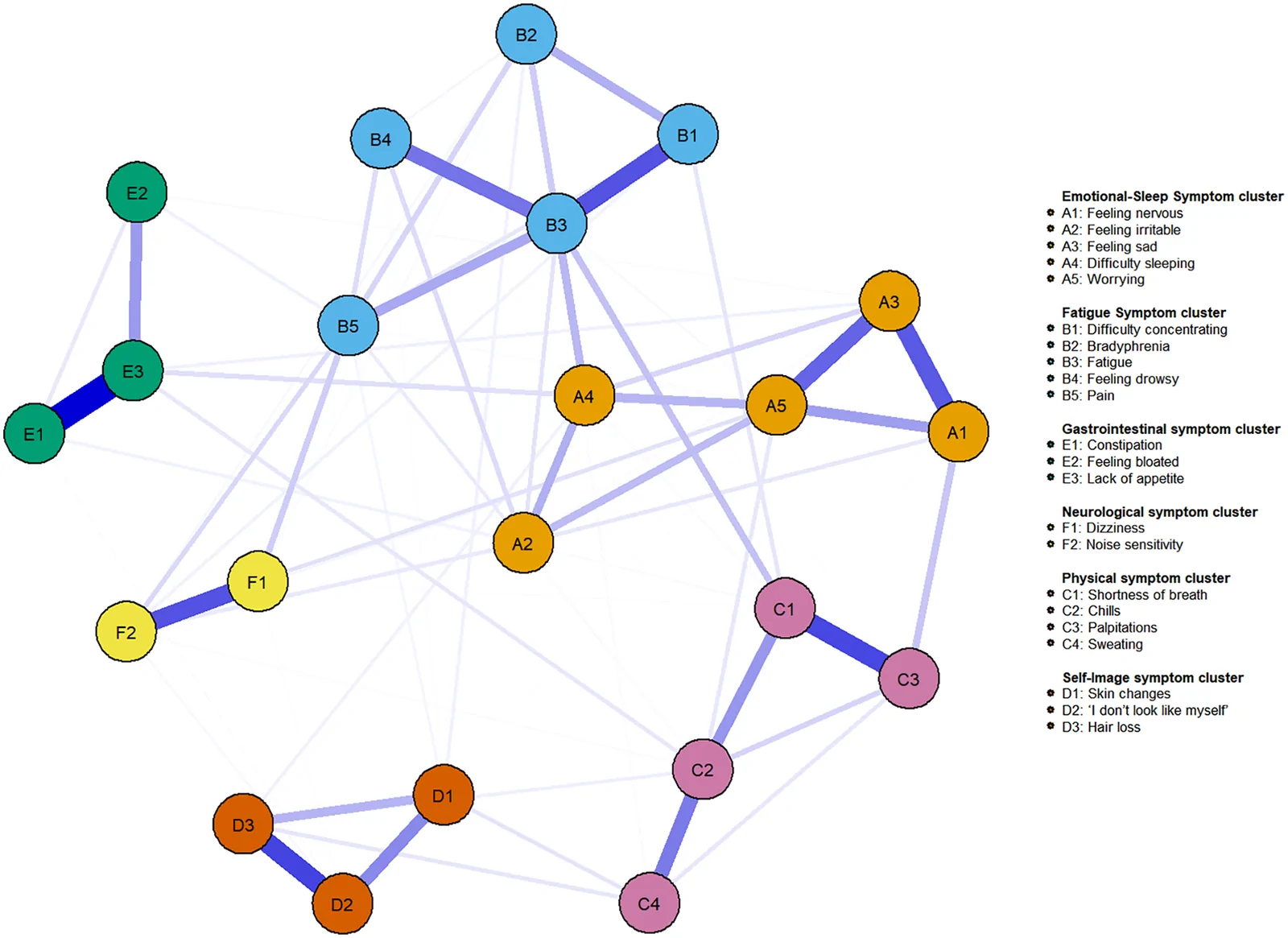
Symptom network of CFS patients. “Nodes” represent symptoms, and “edges” represent the relationships between symptoms. Red edges indicate negative correlations, while blue edges indicate positive correlations. Thicker edges and darker colors signify stronger correlations between nodes, whereas thinner edges, and lighter colors signify weaker correlations between nodes.

**Figure 2:**
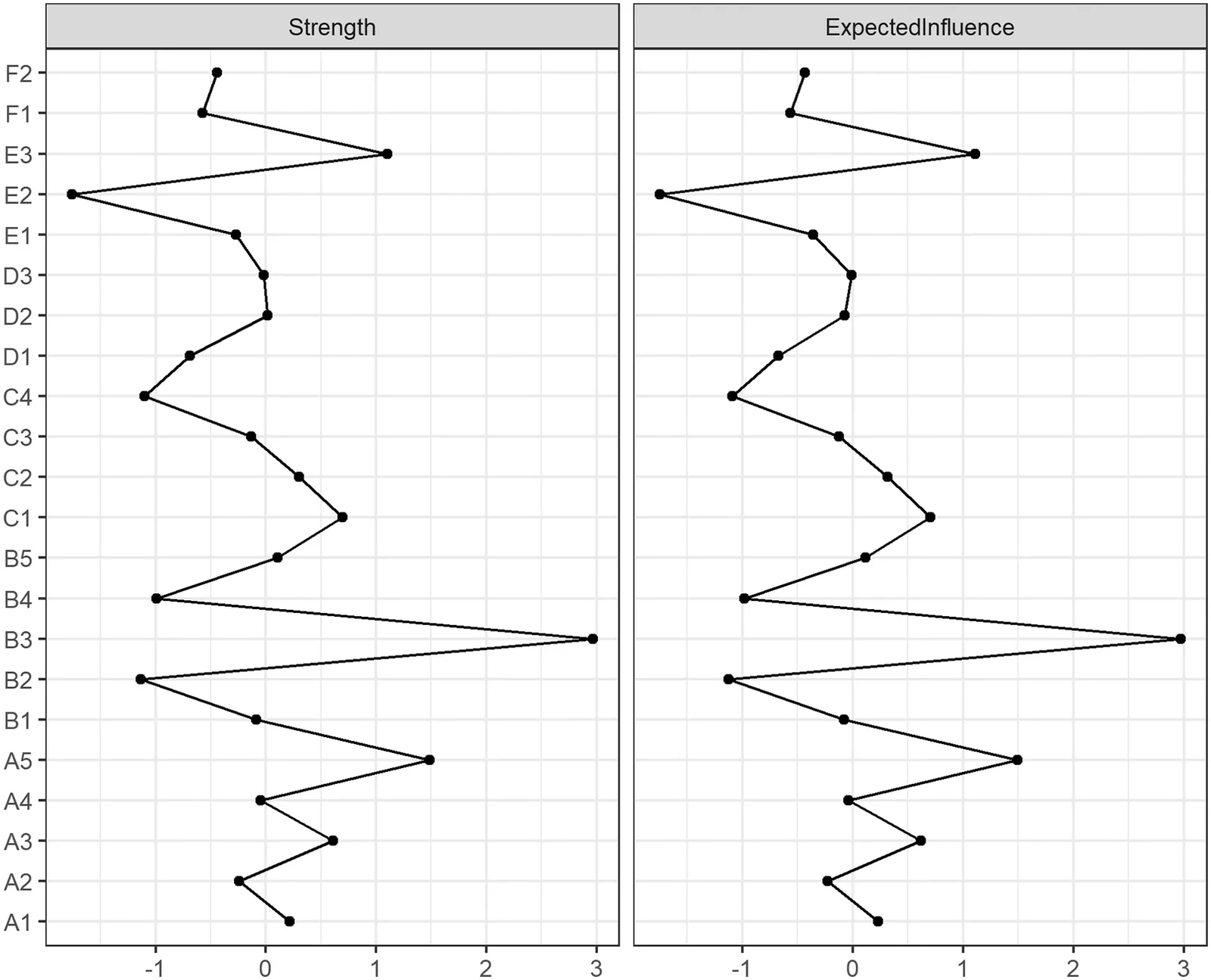
Centrality indicators of symptoms’ network nodes. A1, Feeling nervous; A2, feeling irritable; A3, feeling sad; A4, difficulty sleeping; A5, worrying; B1, difficulty concentrating; B2, bradyphrenia; B3, fatigue; B4, feeling drowsy; B5, pain; C1, shortness of breath; C2, chills; C3, palpitations; C4, sweating; D1, skin changes; D2, “I don’t look like myself”; D3, hair loss; E1, constipation; E2, feeling bloated; E3, lack of appetite; F1, dizziness; F2, noise sensitivity.

**Figure 3:**
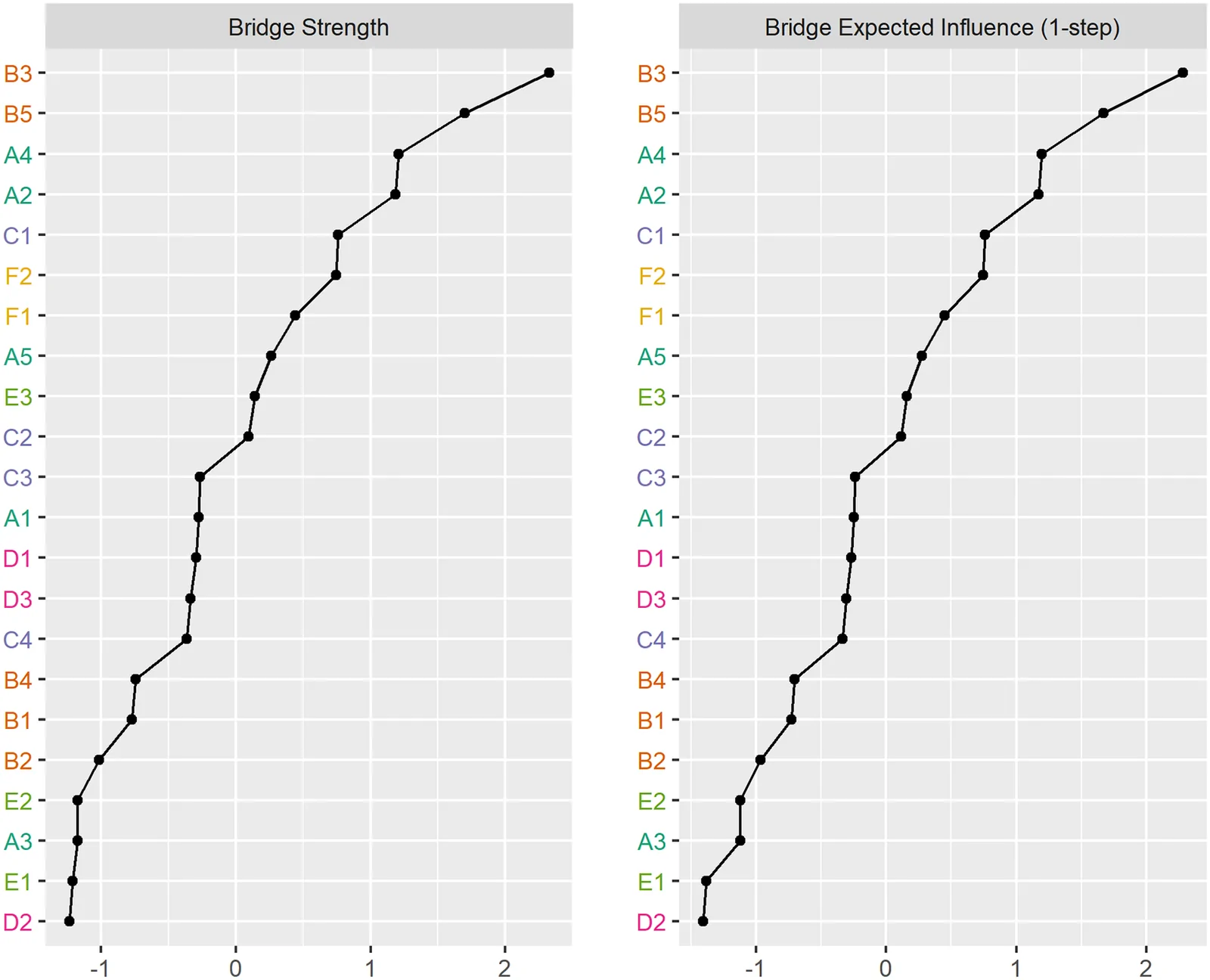
Bridge centrality indicators of symptoms’ network nodes. A1, Feeling nervous; A2, feeling irritable; A3, feeling sad; A4, difficulty sleeping; A5, worrying; B1, difficulty concentrating; B2, bradyphrenia; B3, fatigue; B4, feeling drowsy; B5, pain; C1, shortness of breath; C2, chills; C3, palpitations; C4, sweating; D1, skin changes; D2, “I don’t look like myself”; D3, hair loss; E1, constipation; E2, feeling bloated; E3, lack of appetite; F1, dizziness; F2, noise sensitivity.

### 3.5 Stability of symptom network centrality indices

The grey area in Figure 4 represents the 95% confidence interval (CI) of edge weights. The relatively narrow CI ranges suggest that the estimated edge weights showed excellent precision. The CS coefficients for strength, expected influence, bridge strength, and bridge expected influence were 0.593, 0.593, 0.285, and 0.360, respectively. Strength and expected influence demonstrated excellent stability, whereas bridge strength and bridge expected influence showed acceptable stability. Overall, the major centrality and bridge centrality indices exhibited satisfactory stability and could be interpreted with reasonable confidence. The results for centrality stability are presented in Figure 5. Bootstrap difference tests for node centrality, bridge centrality, and edge weights are presented in Supplementary Figures S6a–e.

**Figure 4:**
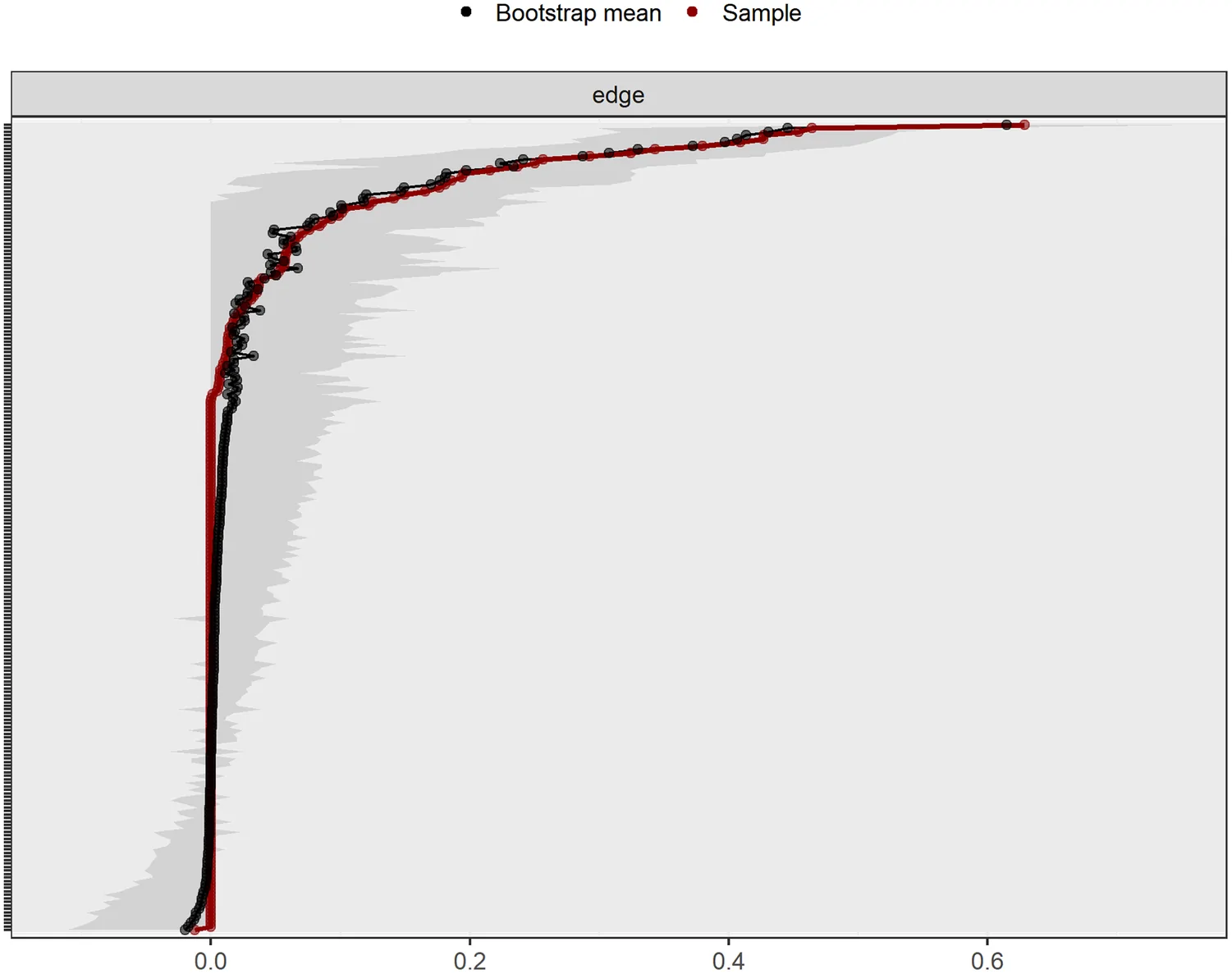
Bootstrap analysis results of the edge weights. The black dots represent the estimated edge weights, and the gray areas represent the 95% confidence intervals derived from bootstrap resampling. Smaller confidence intervals indicate greater accuracy and reliability of the estimated edge weights.

**Figure 5:**
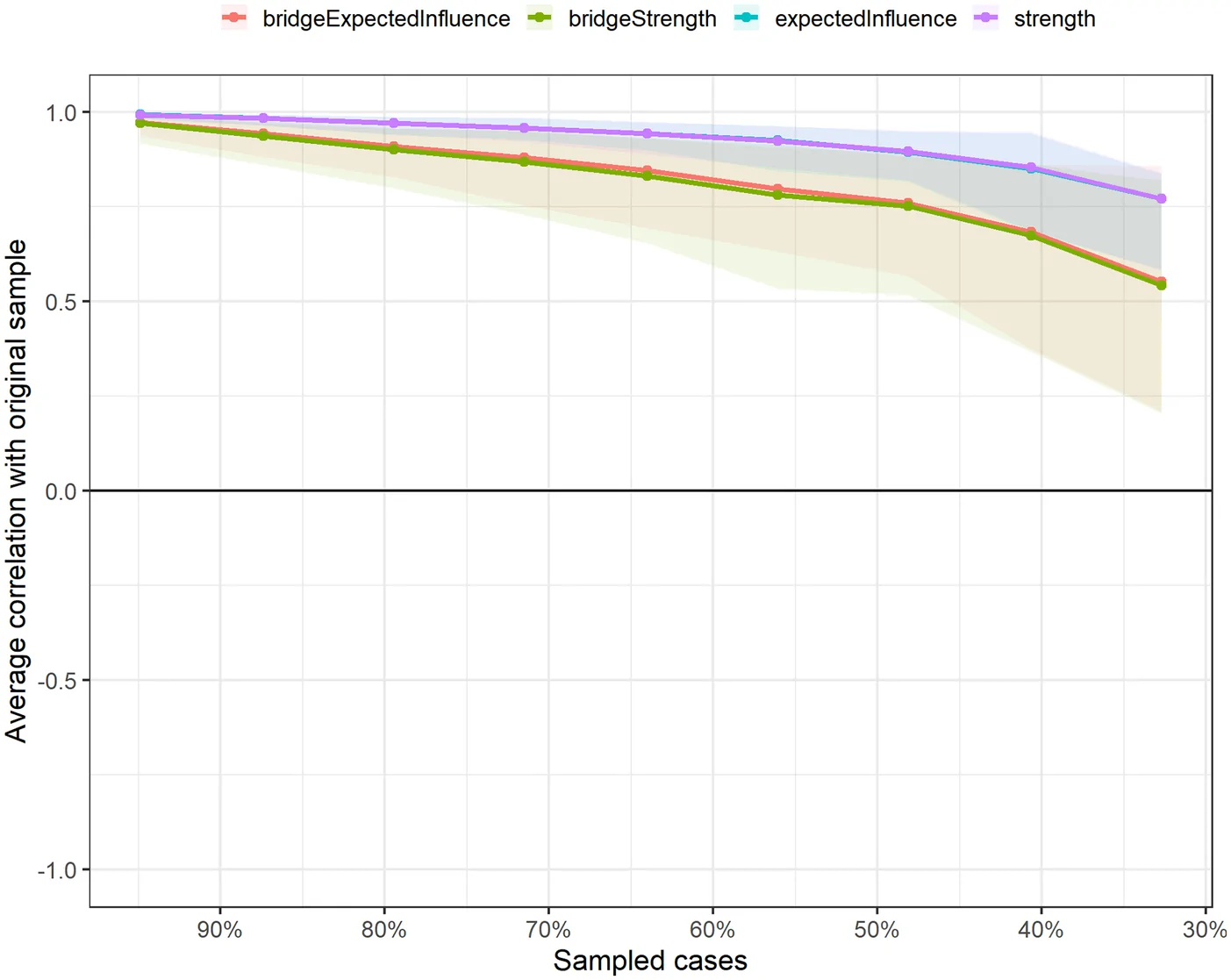
Related stability analysis of symptom network. The curves represent the average correlations between centrality estimates obtained from subsets of the data and those obtained from the original sample for strength, expected influence, bridge strength, and bridge expected influence. The shaded areas represent the corresponding 95% confidence intervals.

### 3.6 Additional analysis

The sensitivity analysis examining the symptom network after adjustment for age and sex was generally consistent with the original analysis. Edge weights were highly similar across the original cor_auto network, the unadjusted Pearson network, and the age- and sex-adjusted Pearson network, with Pearson correlations ranging from 0.978 to 0.993. Expected influence and one-step bridge expected influence rankings were also strongly correlated, with Spearman *p* values ranging from 0.905 to 0.965 and from 0.820 to 0.913, respectively. However, some weak edges, especially low-weight cross-community edges, dropped to zero after adjustment, resulting in a slightly sparser adjusted network. Fatigue and pain retained the highest and second-highest bridge EI, respectively, across the three networks. Detailed results are presented in Supplementary Tables S7.1a–S7.4. The redundancy screening flagged eight of the 231 symptom pairs for item-content review. All 22 symptoms were retained in the primary network after considering their content. Across the 13 single-node-removal models, EI and bridge EI rank correlations with the primary network ranged from 0.914 to 0.997 and from 0.934 to 0.997, respectively. Fatigue ranked first in EI in all models and first in bridge EI in 12 models; its bridge EI ranked third when difficulty sleeping was removed. Removing feeling drowsy left fatigue ranked first in both indices. Detailed results are presented in Supplementary Tables S8b,c.

## 4 Discussion

### 4.1 Strengthening the systematic assessment and management of the fatigue symptom cluster

The results of this study show that a total of 19 symptoms had a prevalence rate greater than 50%, indicating that CFS patients bear a heavy burden of physical and psychological symptoms. The top three symptoms by prevalence were Fatigue (100%), Difficulty concentrating (90.7%), and Feeling drowsy (86.9%), all of which fall under the fatigue symptom cluster, suggesting that this cluster represents one of the most prominent symptom burdens for CFS patients. Fatigue in CFS patients is a persistent and distressing subjective sensation of exhaustion or weariness. Furthermore, since fatigue and cognitive decline may share common pathophysiological mechanisms (10), CFS patients with high levels of fatigue are more likely to experience declines in multiple cognitive domains, such as memory and attention (11). Cognitive impairments, such as memory loss, difficulty concentrating, and bradyphrenia, are common subjective experiences among patients with CFS (12). Compared to physical symptoms, cognitive and psychological symptoms can have a more severe impact on patients' social lives and work (13); studies indicate that patients with CFS often experience difficulties initiating and maintaining sleep, primarily manifested as prolonged sleep latency, increased time spent awake after falling asleep, fragmented sleep, and reduced sleep efficiency; however, changes in total sleep time vary across different studies and age groups. Even with relatively adequate sleep duration, patients often feel that their sleep is not restorative; morning fatigue is not effectively alleviated, and some patients also experience significant daytime sleepiness (14, 15).

At the same time, individuals with CFS may exhibit cognitive impairments such as sustained attention deficits, slowed information processing, and prolonged reaction times; however, current evidence is insufficient to conclude that these cognitive changes are entirely attributable to daytime sleepiness (16). Sleep deprivation and somnolence can further impair alertness and psychomotor responsiveness, thereby potentially increasing safety risks during driving, operating machinery, and other activities requiring sustained attention (17). Furthermore, persistent fatigue, cognitive impairment, and overall functional limitations may collectively restrict patients' social activities, reduce their level of social participation, and increase the likelihood of social isolation or withdrawal (18).

Therefore, clinical assessments should not merely ask patients “whether they feel worn out”; they should also document the severity of fatigue, whether it worsens after activity, the time required for recovery, and the impact of attention, processing speed, and daytime sleepiness on work, study, and daily safety. For patients with a high symptom burden, symptom diaries, activity-rest logs, and tiered follow-up can be used to identify symptom fluctuations and triggers, providing a basis for individualized energy management and supportive interventions.

### 4.2 Emphasizing the coordinated management of the emotional-sleep symptom cluster

This study identified six symptom clusters in CFS patients: the emotional-sleep cluster, the fatigue cluster, the physical cluster, the self-image cluster, the neurological cluster, and the gastrointestinal cluster. Among these, the emotional-sleep cluster includes Worrying (A5, rEI = 1.169, rbEI = 0.206) and Difficulty sleeping (A4, rEI = 0.784, rbEI = 0.309). Worrying had the second-highest EI in the network, while difficulty sleeping was among the symptoms with the highest bridge EI, highlighting the relevance of both symptoms to the assessment of this cluster. Difficulty sleeping is a common and distressing symptom among CFS patients; in this study, its prevalence reached 60.3%, consistent with previous findings that CFS patients commonly experience sleep disturbances and non-restorative sleep (15). Poor sleep quality is associated with lower quality of life (19) and greater emotional distress (20, 21). Work-related and daily-life stress, together with negative life events, may also be relevant to patients' emotional and sleep complaints (22, 23). Previous studies have reported associations between emotional distress and sleep problems, including difficulty falling asleep and early awakening, with anxiety and depressive symptoms frequently accompanying poor sleep quality (24). Research by Guimond et al. (25) also highlights the relevance of difficulties in emotional regulation to the co-occurrence of psychological symptoms. Therefore, healthcare professionals should assess emotional distress and sleep difficulties together, paying particular attention to worrying, sleep patterns, and their impact on daily functioning. For patients reporting sleep difficulties, assessment should also include accompanying emotional symptoms; similarly, sleep complaints should be explored when patients report persistent worrying or emotional distress. For patients with mild to moderate emotional distress or sleep problems, non-pharmacological measures—such as sleep hygiene education, relaxation training, emotional regulation support, and cognitive-behavioral support—may be considered according to individual needs, preferences, and physical tolerance, with the aim of relieving distress and supporting sleep management.

### 4.3 Prioritizing fatigue management and addressing post-exertional malaise

This study found that Fatigue (B3, rEI = 1.541, rbEI = 0.430) showed the highest expected influence and bridge expected influence in the symptom network. Not only does it form a fatigue-related symptom cluster together with difficulty concentrating, bradyphrenia, feeling drowsy, and pain, but it also shows connections with symptoms in other clusters. In the node-removal sensitivity analyses, fatigue consistently ranked first in expected influence and remained among the three highest-ranked symptoms in bridge expected influence. The primary symptom of CFS is fatigue lasting for 6 months or longer; the persistence or recurrence of fatigue severely impairs patients' normal work, education, social behavior, and personal activities, leading to a dramatic decline in their quality of life and health status (26, 27). At the same time, fatigue is associated with a variety of factors. Research indicates that fatigue may be linked to viral infections (28), abnormalities in neuroendocrine regulation, a persistent inflammatory state, immune dysfunction (29), abnormalities in the hypothalamic-pituitary-adrenal axis (30), overexertion, and psychological stress; furthermore, patients' symptoms may persist over the long term and exhibit fluctuations or recurrent exacerbations. Some patients with CFS may experience stigma related to their symptoms, and the severity of their fatigue may not be fully recognized (31, 32). However, the impact of fatigue on patients extends throughout the entire disease course, leaving them in a prolonged state of reduced exercise tolerance, impaired role functioning, and limited social participation. Although fatigue has a high prevalence and significant impact among CFS patients, there remain considerable differences in the assessment tools and evaluation dimensions currently used both domestically and internationally. Assessment should cover fatigue severity, post-exertional malaise, exercise tolerance, and daily functioning. Future studies should use validated ME/CFS-specific assessment tools to strengthen the assessment and identification of these symptoms (33, 34). At the same time, healthcare professionals should prioritize fatigue symptoms in CFS patients. When managing fatigue clinically, healthcare professionals should assess PEM—including its delayed onset, duration, and recovery patterns—to help patients identify their personal energy limits (35). Key intervention strategies may include disease education, individualized energy management, balancing activity and rest, symptom and activity diaries, adjustments to work or study schedules, and symptomatic treatment for sleep, pain, and emotional issues (4). Activity-related advice should be individualized and remain within the patient's energy limits. Programmes involving fixed incremental increases in physical activity or exercise should not be offered. Whether interventions addressing fatigue are associated with improvements in other symptoms requires evaluation in longitudinal and interventional studies.

### 4.4 Conducting systematic pain assessment and dynamic management

This study found that Pain ranked second in terms of bridge expected influence (B5, rbEI = 0.363), making it a relatively important bridge symptom among the symptom clusters of CFS patients. Its relatively high bridge expected influence suggests that pain is an important connecting symptom across the symptom clusters. Previous studies have shown (36) that CFS patients often experience varying degrees of pain, including muscle pain, joint pain, and headaches, the mechanisms of which may be related to chronic low-grade inflammation, central sensitization, and neuroendocrine dysfunction (37, 38). Persistent pain has been associated with an increased stress burden, impaired sleep quality, and more severe fatigue, attention difficulties, and mood symptoms (39–41). At the same time, chronic fatigue may be accompanied by reduced pain tolerance and increased pain sensitivity (42). Therefore, pain may not only be a significant component of the symptom burden in CFS patients but also a key node connecting multiple symptom domains. Furthermore, this study found that Fatigue is the symptom with the highest bridge expected influence in the network (B3, rbEI = 0.430), while pain ranks second, indicating that both symptoms have prominent cross-cluster connections. The coexistence of pain and fatigue also deserves attention when assessing patients' overall symptom experience. Consequently, in the clinical management of CFS, in addition to focusing on core fatigue symptoms, attention should also be given to the systematic assessment and dynamic management of pain symptoms. Comprehensive measures such as pain education, individualized activity management, sleep management, and psychobehavioral interventions may be considered to relieve pain-related symptoms and improve patients' quality of life (4).

### 4.5 Sample characteristics and applicability of the findings

The sample was recruited by convenience sampling from a Traditional Chinese Medicine internal medicine outpatient clinic at a single tertiary hospital. Patients attending this clinic may differ from the broader CFS population in healthcare access, treatment preferences, and functional status. In particular, patients whose symptoms or access barriers prevented outpatient attendance may have been underrepresented. Previous population-based research has documented barriers to healthcare utilization among people with CFS (43). Selection based on the ability to attend outpatient care may therefore have influenced the range of symptom burden captured in this study.

The largest age group in this study was 31–40 years, accounting for 43.5% of participants. This age range is close to the adult peak in recorded CFS incidence at ages 30–39 years reported in a Norwegian population-based registry study (44), providing epidemiological context for the observed age distribution. Participants also predominantly had a college education or above. The high proportion of participants with higher educational attainment may partly reflect differences in access to health information, symptom recognition, and willingness to seek care or participate in research. These education-related differences may influence which symptoms are recognized and reported, while the limited representation of older and less educated patients may leave their symptom experiences less fully captured. Consequently, the observed symptom frequencies and network associations may vary in populations with a different demographic composition.

## 5 Limitations

This study has the following limitations. First, this study employed single-center convenience sampling, with the sample primarily drawn from the Traditional Chinese Medicine Internal Medicine Outpatient Clinic at a Grade A tertiary hospital in Hangzhou. The sample was relatively homogeneous in age and educational attainment. This may restrict the applicability of the findings to older patients, those with lower educational attainment, and patients in other care settings. Future studies should recruit patients from multiple centers and more diverse demographic backgrounds to improve the representativeness of the sample. Second, as a cross-sectional study, this research cannot reveal changes in patients' symptom networks over time nor determine causal relationships between symptoms. Future studies should use longitudinal network analyses to examine changes in symptom clusters and network structure over time. Third, symptom assessments in this study were based on self-reported data, which may introduce some bias. Future research could collect data using ecological momentary assessment methods to provide a basis for implementing precision symptom cluster management. Fourth, participants were identified using the 1994 Fukuda criteria, which do not require post-exertional malaise for diagnosis. The symptom assessment also did not specifically characterize post-exertional malaise or orthostatic intolerance. Consequently, the findings may not generalize to populations meeting the 2015 Institute of Medicine criteria for ME/CFS (45). Future studies should use the 2015 Institute of Medicine criteria and validated ME/CFS-specific assessment tools. Fifth, the adapted MSAS has not undergone formal validation in patients with CFS. Although the adaptation was informed by a literature review, patient interviews, and research-team discussion, the reported internal consistency and exploratory factor analysis provide only preliminary psychometric evidence. Incomplete symptom coverage and potential item overlap may have influenced the identified clusters and centrality estimates. Future studies should use validated ME/CFS-specific instruments and evaluate the adapted symptom set in independent samples. Sixth, the sensitivity analysis examined adjustment for age and sex only. The influence of other demographic and clinical characteristics on the estimated network remains to be assessed in larger studies.

## 6 Implications for clinical practice

This study combines symptom cluster identification with symptom network analysis, supporting a shift in symptom management in CFS patients from a “single-symptom approach” to “coordinated management of symptom clusters.” Clinicians can use a simplified symptom checklist during initial consultations and follow-up visits to prioritize screening for fatigue, difficulty concentrating, feeling drowsy, worrying, difficulty sleeping, and pain; clinical assessment should also include post-exertional malaise and orthostatic intolerance, types of sleep disturbances, and pain characteristics. The observed symptom patterns may inform a structured assessment that considers fatigue, mood, sleep, and pain together. Fatigue may serve as an initial assessment focus, with individualized energy management and an appropriate balance between activity and rest. Mood screening, sleep assessment, and pain management can be conducted concurrently, with multidisciplinary collaboration tailored to the patient's functional status, life goals, and preferences. During follow-up, symptom diaries or digital tools can be used to dynamically record symptom intensity, activity levels, and triggers for symptom exacerbation, enabling timely adjustments to the intensity of interventions. It is important to emphasize that network centrality can only suggest candidate targets for clinical assessment and intervention prioritization; it cannot replace etiological evaluation, differential diagnosis, or individualized clinical decision-making.

## 7 Conclusion

This study surveyed 214 patients with CFS and identified six symptom clusters: emotional-sleep, fatigue, physical, self-image, gastrointestinal, and neurological. These clusters accounted for 63.114% of the total variance. Fatigue had the highest estimated expected influence and bridge expected influence. Worrying and “lack of appetite” also had relatively high expected influence, while pain and difficulty sleeping showed relatively high bridge expected influence. The results indicate that the symptom burden in CFS patients is multidimensional and interconnected. These exploratory findings may inform symptom assessment in similar clinical settings, but broader applicability and treatment implications require further validation. Future research should validate the structure of symptom clusters and network characteristics through multicenter, longitudinal studies and independent samples and further evaluate whether management strategies targeting core and bridge symptoms can improve patient outcomes.

## Data Availability

The raw data supporting the conclusions of this article will be made available by the authors, without undue reservation.
