# Supplementary material for: Symptom clusters and symptom network characteristics in chronic fatigue syndrome patients: a cross-sectional study
Source: Front Med (Lausanne). 2026 Oct 20;13:1955021. doi: 10.3389/fmed.2026.1955021 (PMC13727641; doi:10.3389/fmed.2026.1955021)
Supplement: Supplementary file 1 [file Datasheet1.pdf]

Supplementary Material

Index of supplementary materials

Supplementary Material 1. Symptom selection and adaptation of the MSAS

Table S1. Sources, interview evidence, and selection decisions

Supplementary Material 2. Exploratory factor analysis of the adapted MSAS

Table S2.1. Common-factor parallel analysis

Table S2.2. Promax pattern matrix and communalities

Table S2.3. Correlations between the eight rotated factors

Supplementary Material 3. Symptom prevalence, frequency, severity, and distress

Table S3. Symptom prevalence, frequency, severity, and distress

Supplementary Material 4. PCA-derived symptom clusters

Table S4. Rotated component matrix

Supplementary Material 5. Centrality and bridge centrality indices

Table S5. Centrality and bridge centrality indices

Supplementary Material 6. Bootstrap difference tests of network indices

Figure S6a. Bootstrap difference test of node strength

Figure S6b. Bootstrap difference test of node expected influence

Figure S6c. Bootstrap difference test of bridge strength

Figure S6d. Bootstrap difference test of bridge expected influence

Figure S6e. Bootstrap difference test of edge weights

Supplementary Material 7. Age- and sex-adjusted network sensitivity analysis

Table S7.1a. Characteristics of the three symptom networks

Table S7.1b. Pairwise comparisons of network characteristics

Table S7.2. Expected influence values and rankings across the three networks

Table S7.3. One-step bridge expected influence values and rankings across the three networks

Table S7.4. Ten symptom pairs with the largest absolute changes in edge weights after adjustment

Supplementary Material 8. Community assignments, redundancy screening, and node-removal sensitivity analyses

Table S8a. Node-to-community assignment

Table S8b. Screening of all 231 symptom pairs

Table S8c. Single-node-removal sensitivity analyses

Supplementary Material 1. Symptom selection and adaptation of the MSAS

Table S1. Sources, interview evidence and selection decisions

Symptom or item	Source	Refs.	Summary of patient feedback	Team rationale	Final decision
**Bradyphrenia**	Literature; DSQ-2; interviews	[2–4]	008 explicitly described slower thinking and speech; 011 described intermittent slowing of thinking, speech, and expression when tired. 007 described being unable to think effectively after prolonged document work; 002 described less coherent thinking. Broader attention or reading difficulties also occurred. 002 and 009 also gave spontaneous descriptions of confusion or brain fog.	Provides a specific description of slowed thinking alongside the existing MSAS concentration item. The symptom content is supported by the literature and interview extracts.	Added
**Chills**	Literature; DSQ-2; interviews	[2,3]	003 described cold sensations and cold limbs during a standing episode; the participant linked some temperature changes to menstrual or hormonal fluctuations. 008 described cold sensations when unwell. 004 reported sensitivity to cold. 010 reported intolerance of cold air-conditioning. 011 described cold sweating.	Adds a cold-related symptom absent from the original MSAS.	Added

Symptom or item	Source	Refs.	Summary of patient feedback	Team rationale	Final decision
**Noise sensitivity**	Literature; DSQ-2; interviews	[2,3]	007 described annoyance at keyboard sounds and waking with slight noise. 008 reported annoyance when sounds were excessively loud. 010 described waking with slight sounds or a spouse moving. 002 described light sleep and easy awakening while stating that noise was otherwise tolerable. These reports were prompted.	Adds an auditory symptom absent from the original MSAS.	Added
**Palpitations**	Literature; DSQ-2; interviews	[2,3]	003 described palpitations while seated and pounding heartbeat during standing handover. 005 reported occasional mild palpitations. 011 spontaneously described a rapid, pounding heartbeat while seated, aggravated by frustration or anger. 002 described a racing heartbeat on standing. For 007, the extraction record documents endorsement after audio review, although the supplied transcript excerpt contains no explicit affirmative answer.	Captures a subjective heartbeat symptom absent from the original MSAS.	Added
**Orthostatic symptoms**	Literature; DSQ-2; interviews	[2,3]	002 described a racing heartbeat, blurred vision, breathlessness and limb weakness after standing, relieved by sitting. 003 described shaking, cold limbs and pounding heartbeat during standing handover. 011 described occasional dizziness or visual darkening after rising and needing support or rest during prolonged standing.	The final scale includes dizziness and palpitations.	Not added as a separate item

Symptom or item	Source	Refs.	Summary of patient feedback	Team rationale	Final decision
**Sore throat, flu-like symptoms**	Literature; DSQ-2; interviews	[2,3]	002 described recurrent dry, painful throat and flu-like episodes without apparent lymph-node swelling. 007 described roughly monthly throat pain unrelated to a cold and denied lymph-node symptoms and low fever. 008 reported recurrent throat pain, and episodes described as colds.	Pain was assessed using the existing MSAS item; sore throat and flu-like symptoms were not assessed separately.	Not added separately
**Disorientation, poor coordination and difficulty focusing vision**	Literature; DSQ-2; interviews	[2,4]	002 described lifelong poor direction sense and coordination without a change. 010 reported poor direction sense without an established onset. 011 described difficulty naming compass directions while retaining route finding and normal walking coordination.	Interview reports did not clearly indicate illness-related changes in sense of direction or coordination.	Not added separately
**Alcohol intolerance**	Literature; DSQ-2; interviews	[2,3]	011 reported being able to drink a little but becoming intoxicated easily. This describes alcohol sensitivity without establishing a new illness-related intolerance.	Lack of specificity in symptom definition	Not added
**Medication intolerance**	Literature; DSQ-2; interviews	[2,3]	008 reported cephalosporin allergy; 011 reported no known medication allergy.	The interviews did not establish a broader pattern of medication intolerance. Allergy and nonspecific intolerance were not pooled as the same finding.	Not added
**Problems with sexual interest or activity**	Original MSAS; preliminary survey	[1]	Patients avoided or declined this item because of privacy concerns during the preliminary survey.	The item was removed because participants avoided or declined to answer it owing to privacy concerns during the preliminary survey.	Removed

Abbreviations: MSAS, Memorial Symptom Assessment Scale; DSQ-2, DePaul Symptom Questionnaire-2; CFS, chronic fatigue syndrome. No full DSQ-2 administration or DSQ-2 score is implied by its use as an item source.

References and source records

[1] Portenoy RK, Thaler HT, Kornblith AB, et al. The Memorial Symptom Assessment Scale: an instrument for the evaluation of symptom prevalence, characteristics and distress. Eur J Cancer. 1994;30A(9):1326–1336. doi:10.1016/0959-8049(94)90182-1.

[2] Bedree H, Sunnquist M, Jason LA. The DePaul Symptom Questionnaire-2: a validation study. Fatigue.

2019;7(3):166–179. doi:10.1080/21641846.2019.1653471.
[3] Tschopp R, König RS, Rejmer P, Paris DH. Myalgic encephalomyelitis/chronic fatigue syndrome (ME/CFS): a preliminary survey among patients in Switzerland. Heliyon. 2023;9(5):e15595. doi:10.1016/j.heliyon.2023.e15595.
[4] Jain V, Arunkumar A, Kingdon C, Lacerda E, Nacul L. Prevalence of and risk factors for severe cognitive and sleep symptoms in ME/CFS and MS. BMC Neurol. 2017;17:117. doi:10.1186/s12883-017-0896-0.

Supplementary Material 2. Exploratory factor analysis of the adapted MSAS

2.1. Common-factor parallel analysis

Table S2.1. Common-factor parallel analysis for the first ten ordered eigenvalues

Order	Observed eigenvalue	Permutation 95th percentile	Exceeds reference
1	5.285	1.145	Yes
2	1.898	0.985	Yes
3	1.682	0.886	Yes
4	1.359	0.803	Yes
5	1.068	0.735	Yes
6	0.898	0.670	Yes
7	0.836	0.605	Yes
8	0.599	0.550	Yes
9	0.453	0.499	No
10	0.390	0.449	No

2.2. Complete Promax pattern matrix and communalities

Table S2.2. Complete Promax pattern matrix and communalities for the 34 severity items

Symptom	h^2	F1	F2	F3	F4	F5	F6	F7	F8
Feeling nervous	0.654	**0.855**	-0.074	-0.112	0.013	0.141	-0.093	-0.041	0.028
Feeling irritable	0.353	**0.431**	0.201	0.067	-0.030	-0.019	0.132	-0.016	0.004
Feeling sad	0.650	**0.836**	-0.060	-0.046	0.025	-0.001	-0.063	0.039	-0.053

Difficulty sleeping	0.454	0.350	0.182	0.109	0.033	-0.005	0.055	0.212	-0.223
Worrying	0.734	**0.833**	0.008	-0.039	0.028	-0.062	0.092	0.044	-0.059
Difficulty concentrating	0.553	-0.075	**0.681**	0.088	0.034	0.101	0.061	0.015	-0.119
**Bradyphrenia**	0.332	-0.101	**0.578**	0.025	0.046	0.039	0.056	-0.053	0.125
Fatigue	0.746	0.056	**0.816**	0.070	0.019	0.115	-0.046	-0.020	0.028
Feeling drowsy	0.392	0.135	**0.580**	-0.052	0.038	0.028	-0.073	-0.038	0.255
Pain	0.455	0.017	**0.523**	0.016	0.017	-0.035	0.224	0.116	0.109
Shortness of breath	0.514	0.093	0.187	-0.014	-0.173	**0.639**	-0.030	0.064	0.014
**Chills**	0.454	0.012	0.039	0.115	-0.024	**0.654**	0.039	-0.097	-0.060
**Palpitations**	0.465	0.177	0.007	-0.067	-0.146	**0.675**	-0.073	0.013	0.059
Sweating	0.302	-0.135	0.093	0.004	0.125	**0.508**	-0.008	-0.027	-0.025
Skin changes	0.405	0.045	0.112	0.015	**0.647**	-0.007	-0.136	-0.018	0.090
I don't look like myself	0.680	0.039	0.051	-0.114	**0.863**	-0.164	0.051	-0.106	0.078
Hair loss	0.547	0.027	-0.077	-0.103	**0.725**	-0.010	0.054	0.018	0.039
Constipation	0.669	-0.115	0.078	**0.911**	-0.053	0.028	-0.120	-0.073	-0.098
Feeling bloated	0.218	-0.007	0.040	**0.414**	0.008	-0.018	-0.029	0.061	0.106
Lack of appetite	0.772	-0.017	0.063	**0.933**	-0.099	0.017	0.019	-0.117	-0.056
Dizziness	0.401	0.069	0.111	-0.098	-0.054	-0.055	**0.641**	-0.004	-0.034
**Noise sensitivity**	0.648	0.040	0.085	0.017	-0.099	-0.077	**0.912**	-0.181	-0.264
Numbness/tingling in hands/feet	0.184	-0.112	0.129	-0.071	0.052	0.072	-0.003	0.371	-0.096
Dry mouth	0.144	0.158	-0.059	0.069	0.055	0.032	0.091	0.172	-0.006
Cough	0.079	-0.077	-0.072	-0.025	-0.012	0.126	0.190	0.054	0.062

Nausea	0.398	0.063	-0.063	-0.099	-0.052	-0.088	-0.004	**0.673**	0.120
Vomiting	0.375	0.078	-0.043	0.067	-0.030	0.002	-0.174	**0.600**	0.076
Change in the way food tastes	0.239	0.152	-0.232	0.236	0.143	0.114	0.050	-0.069	0.104
Mouth sores	0.166	-0.090	-0.010	0.136	-0.019	-0.105	0.284	0.092	0.152
Diarrhea	0.250	0.005	-0.003	-0.007	-0.008	-0.020	0.006	**0.506**	-0.059
Problems with urination	0.260	0.038	0.247	-0.021	-0.052	-0.085	-0.053	-0.060	**0.513**
Weight loss	0.057	0.020	0.007	-0.047	0.073	0.065	0.179	-0.048	0.007
Itching	0.242	-0.046	0.060	0.072	**0.465**	-0.019	-0.058	0.055	-0.173
Swelling of arms or legs	0.254	-0.094	-0.002	0.006	0.068	0.083	-0.069	0.071	**0.484**

h^2, communality. Bold values indicate absolute pattern loadings ≥ 0.40. Loadings are regression-type pattern coefficients, not structure correlations. F1–F8 are descriptive identifiers and are not proposed validated MSAS subscales.

2.3. Correlations between the eight rotated factors

Table S2.3. Correlations between the eight rotated factors

**Factor**	**F1**	**F2**	**F3**	**F4**	**F5**	**F6**	**F7**	**F8**
F1	1.000	0.312	0.406	0.141	0.309	0.302	0.394	0.008
F2	0.312	1.000	0.065	0.068	0.237	0.209	0.271	-0.127
F3	0.406	0.065	1.000	0.175	0.226	0.261	0.378	0.255
F4	0.141	0.068	0.175	1.000	0.360	0.355	0.252	0.098
F5	0.309	0.237	0.226	0.360	1.000	0.406	0.324	0.130
F6	0.302	0.209	0.261	0.355	0.406	1.000	0.342	0.329
F7	0.394	0.271	0.378	0.252	0.324	0.342	1.000	0.044
F8	0.008	-0.127	0.255	0.098	0.130	0.329	0.044	1.000

Supplementary Material 3. Symptom prevalence, frequency, severity, and distress

Table S3. Prevalence, frequency, severity, and distress of symptoms

Symptom	Prevalence (n, %)	Frequency	Severity	Distress
		Mean (SD)	Mean (SD)	Mean (SD)
Difficulty concentrating	194(90.7)	1.93 (0.82)	1.77 (0.81)	1.42 (0.87)
Pain	159(74.3)	1.81 (0.82)	1.74 (0.80)	1.50 (0.83)
Fatigue	214(100)	2.70 (0.87)	1.94 (0.93)	1.44 (1.01)
Cough	72(33.6)	1.40 (0.62)	1.35 (0.61)	0.96 (0.72)
Feeling nervous	155(72.4)	2.16 (0.95)	2.28 (0.99)	1.65 (1.06)
Dry mouth	148(69.2)	1.67 (0.74)	1.53 (0.75)	1.08 (0.81)
Nausea	55(25.7)	1.49 (0.66)	1.29 (0.57)	0.93 (0.74)
Feeling drowsy	186(86.9)	2.16 (0.82)	2.11 (0.79)	1.58 (0.90)
Numbness/tingling in hands/feet	67(31.3)	1.37 (0.55)	1.15 (0.36)	0.94 (0.67)
Difficulty sleeping	129(60.3)	1.77 (0.77)	1.59 (0.74)	2.67 (0.74)
Feeling bloated	123(57.5)	1.60 (0.72)	1.63 (0.65)	1.08 (0.77)
Problems with urination	5(2.3)	1.80 (1.10)	2.20 (0.84)	1.80 (0.84)
Vomiting	13(6.1)	1.38 (0.51)	1.31 (0.48)	1.08 (0.76)
Shortness of breath	99(46.3)	2.25 (0.93)	2.31 (1.00)	1.94 (1.08)
Diarrhea	73(34.1)	1.49 (0.58)	1.58 (0.66)	0.96 (0.79)
Feeling sad	158(73.8)	1.84 (0.77)	1.88 (0.72)	1.37 (0.91)
Sweating	124(57.9)	1.77 (0.87)	1.83 (0.88)	1.28 (0.85)
Worrying	158(73.8)	1.75 (0.74)	1.72 (0.78)	1.51 (0.88)
Itching	65(30.4)	1.68 (0.66)	1.62 (0.63)	1.09 (0.76)
Lack of appetite	112(52.3)	1.69 (0.74)	1.70 (0.77)	1.22 (0.92)

Dizziness	113(52.8)	1.82 (0.75)	1.64 (0.72)	1.19 (0.92)
Difficulty swallowing	0(0.0)	0.00 (0.00)	0.00 (0.00)	0.00 (0.00)
Feeling irritable	144(67.3)	1.68 (0.69)	1.60 (0.61)	1.13 (0.81)
Palpitations	95(44.4)	1.86 (0.87)	1.84 (0.96)	1.54 (0.97)
Bradyphrenia	150(70.1)	1.85 (0.75)	1.77 (0.81)	1.36 (0.86)
Chills	118(55.1)	1.63 (0.62)	1.53 (0.57)	1.13 (0.80)
Noise sensitivity	148(69.2)	1.67 (0.70)	1.67 (0.68)	1.12 (0.80)
Mouth sores	63(29.4)	-	1.43 (0.67)	1.13 (0.73)
Change in the way food tastes	61(28.5)	-	1.36 (0.58)	0.89 (0.71)
Weight loss	26(12.1)	-	1.42 (0.81)	0.81 (0.80)
Hair loss	93(43.5)	-	1.65 (0.73)	1.18 (0.90)
Constipation	109(50.9)	-	1.64 (0.73)	1.20 (0.90)
Swelling of arms or legs	26(12.1)	-	1.23 (0.43)	0.92 (0.80)
'I don't look like myself'	105(49.1)	-	1.59 (0.76)	1.17 (0.88)
Skin changes	131(61.2)	-	1.72 (0.81)	1.18 (0.82)

Abbreviations: SD, standard deviation.

Note: "–" indicates that frequency was not assessed or was not applicable.

Supplementary Material 4. PCA-derived symptom clusters

Table S4. Rotated component matrix from the principal component analysis of symptom clusters

**Symptom**	**Factor load**					
	**Emotional-sleep symptom cluster**	**Fatigue symptom cluster**	**Physical symptom cluster**	**Self-image symptom cluster**	**Gastrointestinal symptom cluster**	**Neurological symptom cluster**

A1 Feeling nervous	0.819	0.013	0.194	0.039	0.022	0.025
A2 Feeling irritable	0.564	0.332	0.02	0.013	0.126	0.111
A3 Feeling sad	0.853	0.024	0.086	0.027	0.085	0.028
A4 Difficulty sleeping	0.578	0.301	0.089	0.067	0.185	0.071
A5 Worrying	0.836	0.117	0.091	0.057	0.09	0.169
B1 Difficulty concentrating	0.09	0.743	0.177	0.011	0.046	0.1
B2 Bradyphrenia	-0.06	0.681	0.049	0.074	0.046	0.129
B3 Fatigue	0.227	0.81	0.188	-0.001	0.06	0.022
B4 Feeling drowsy	0.191	0.678	0.081	0.04	-0.03	-0.049
B5 Pain	0.153	0.62	0.089	0.088	0.096	0.312
C1 Shortness of breath	0.227	0.276	0.692	-0.064	0.04	0.055
C2 Chills	0.103	0.071	0.752	0.095	0.154	0.128
C3 Palpitations	0.241	0.09	0.736	-0.077	-0.026	-0.003
C4 Sweating	-0.095	0.13	0.635	0.24	0.029	0.026
D1 Skin changes	0.071	0.146	0.077	0.765	0.09	-0.086
D2 ‘I don’t look like myself’	0.028	0.043	-0.021	0.835	-0.058	0.12
D3 Hair loss	0.053	-0.026	0.1	0.825	-0.037	0.084
E1 Constipation	0.083	0.057	0.045	-0.063	0.857	-0.061
E2 Feeling bloated	0.074	0.037	0.046	0.083	0.653	0.003
E3 Lack of appetite	0.18	0.063	0.067	-0.055	0.862	0.067
F1 Dizziness	0.128	0.151	0.085	0.061	-0.032	0.819
F2 Noise sensitivity	0.126	0.152	0.069	0.044	0.023	0.832
Cumulative variance contribution	14.044	27.305	37.179	46.647	55.867	63.114

rate (%)						

Supplementary Material 5. Centrality and bridge centrality indices

Table S5. Centrality and bridge centrality indices of symptoms in the symptom network analysis

Node	Strength	Expected Influence	Bridge Strength	Bridge Expected Influence
Feeling nervous	0.8506624	0.8506624	0.14766421	0.14766421
Feeling irritable	0.7358784	0.7358784	0.30644579	0.30644579
Feeling sad	0.9486985	0.9486985	0.04965022	0.04965022
Difficulty sleeping	0.7836861	0.7836861	0.30898986	0.30898986
Worrying	1.1691085	1.1691085	0.20591689	0.20591689
Difficulty concentrating	0.7739469	0.7739469	0.0936239	0.0936239
Bradyphrenia	0.5107175	0.5107175	0.06704605	0.06704605
Fatigue	1.5412463	1.5412463	0.43045941	0.43045941
Feeling drowsy	0.5471027	0.5471027	0.0965373	0.0965373
Pain	0.8226749	0.8226749	0.36253545	0.36253545
Shortness of breath	0.9704983	0.9704983	0.2601924	0.2601924
Chills	0.8722715	0.8722715	0.1879309	0.1879309
Palpitations	0.7625614	0.7625614	0.14840001	0.14840001
Sweating	0.5191399	0.5191399	0.13761631	0.13761631
Skin changes	0.6237407	0.6237407	0.14539748	0.14539748
‘I don’t look like myself’	0.8000296	0.7746696	0.04323542	0.01787547
Hair loss	0.7909765	0.7909765	0.14098347	0.14098347
Constipation	0.7284615	0.7031016	0.04560036	0.02024041
Feeling bloated	0.3540477	0.3540477	0.04978372	0.04978372
Lack of appetite	1.0720732	1.0720732	0.19294556	0.19294556
Dizziness	0.6522675	0.6522675	0.22548025	0.22548025
Noise sensitivity	0.6852996	0.6852996	0.25851232	0.25851232

Supplementary Material 6. Bootstrap difference tests of network indices

Figure S6a. Bootstrap difference test of node strength

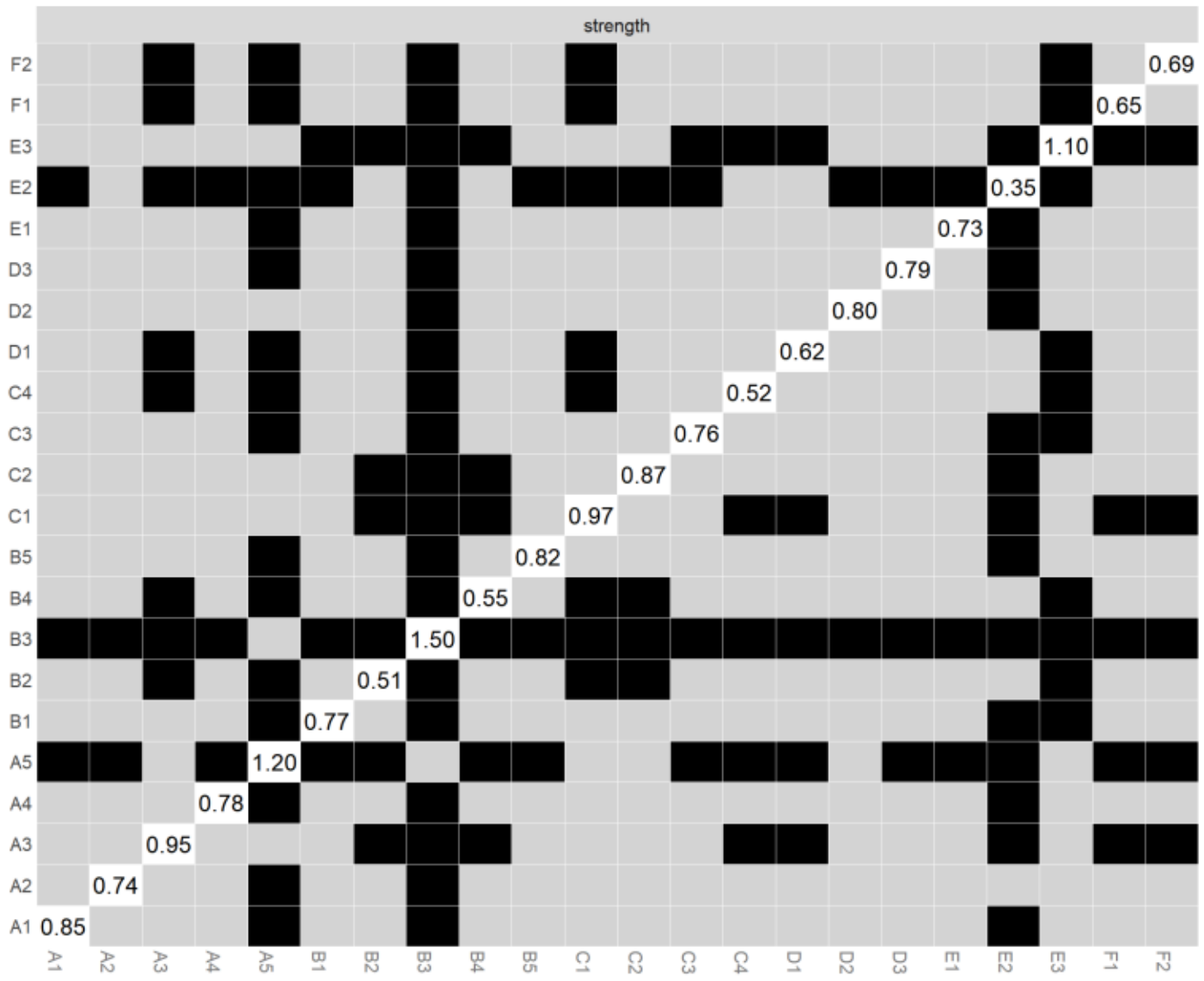

Abbreviations: A1, Feeling nervous; A2, Feeling irritable; A3, Feeling sad; A4, Difficulty sleeping; A5, Worrying; B1, Difficulty concentrating; B2, Bradyphrenia; B3, Fatigue; B4, Feeling drowsy; B5, Pain; C1, Shortness of breath; C2, Chills; C3, Palpitations; C4, Sweating; D1, Skin changes; D2, "I don't look like myself"; D3, Hair loss; E1, Constipation; E2, Feeling bloated; E3, Lack of appetite; F1, Dizziness; F2, Noise sensitivity.

Figure S6b. Bootstrap difference test of node expected influence

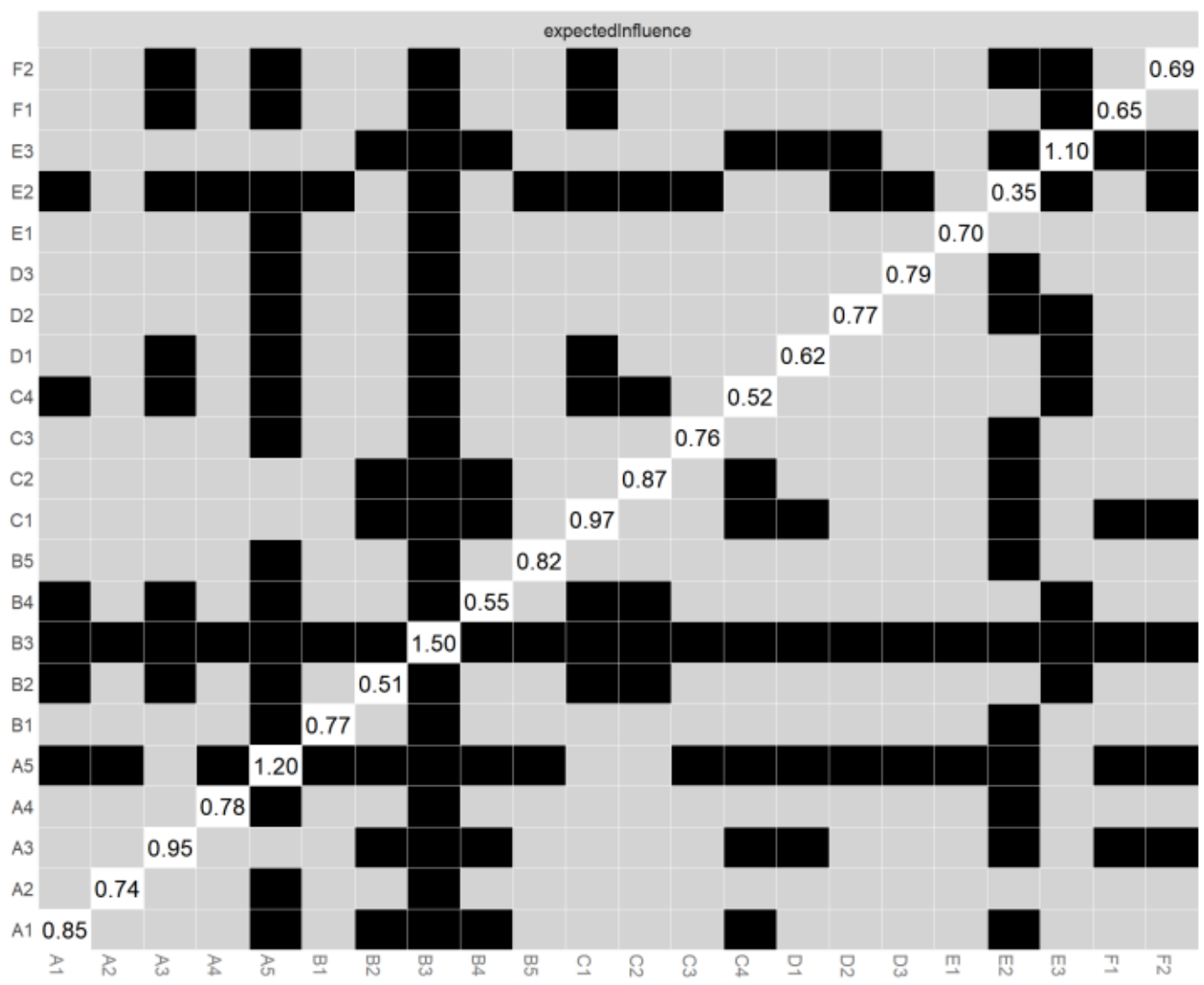

Abbreviations: A1, Feeling nervous; A2, Feeling irritable; A3, Feeling sad; A4, Difficulty sleeping; A5, Worrying; B1, Difficulty concentrating; B2, Bradyphrenia; B3, Fatigue; B4, Feeling drowsy; B5, Pain; C1, Shortness of breath; C2, Chills; C3, Palpitations; C4, Sweating; D1, Skin changes;

D2, “I don’t look like myself”; D3, Hair loss; E1, Constipation; E2, Feeling bloated; E3, Lack of appetite; F1, Dizziness; F2, Noise sensitivity.

Figure S6c. Bootstrap difference test of bridge strength

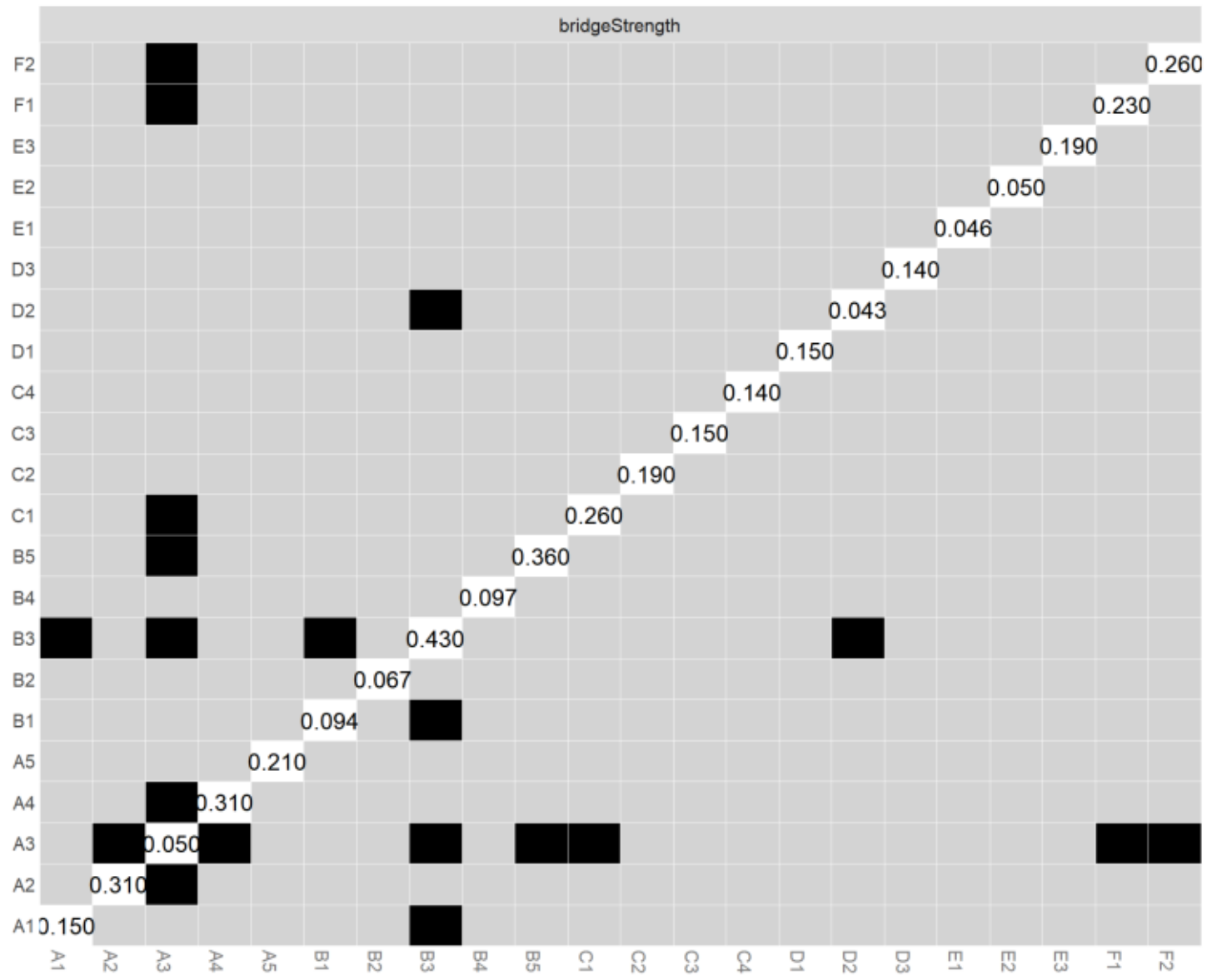

Abbreviations: A1, Feeling nervous; A2, Feeling irritable; A3, Feeling sad; A4, Difficulty sleeping; A5, Worrying; B1, Difficulty concentrating; B2, Bradyphrenia; B3, Fatigue; B4, Feeling drowsy; B5, Pain; C1, Shortness of breath; C2, Chills; C3, Palpitations; C4, Sweating; D1, Skin changes; D2, “I don’t look like myself”; D3, Hair loss; E1, Constipation; E2, Feeling bloated; E3, Lack of appetite; F1, Dizziness; F2, Noise sensitivity.

Figure S6d. Bootstrap difference test of bridge expected influence

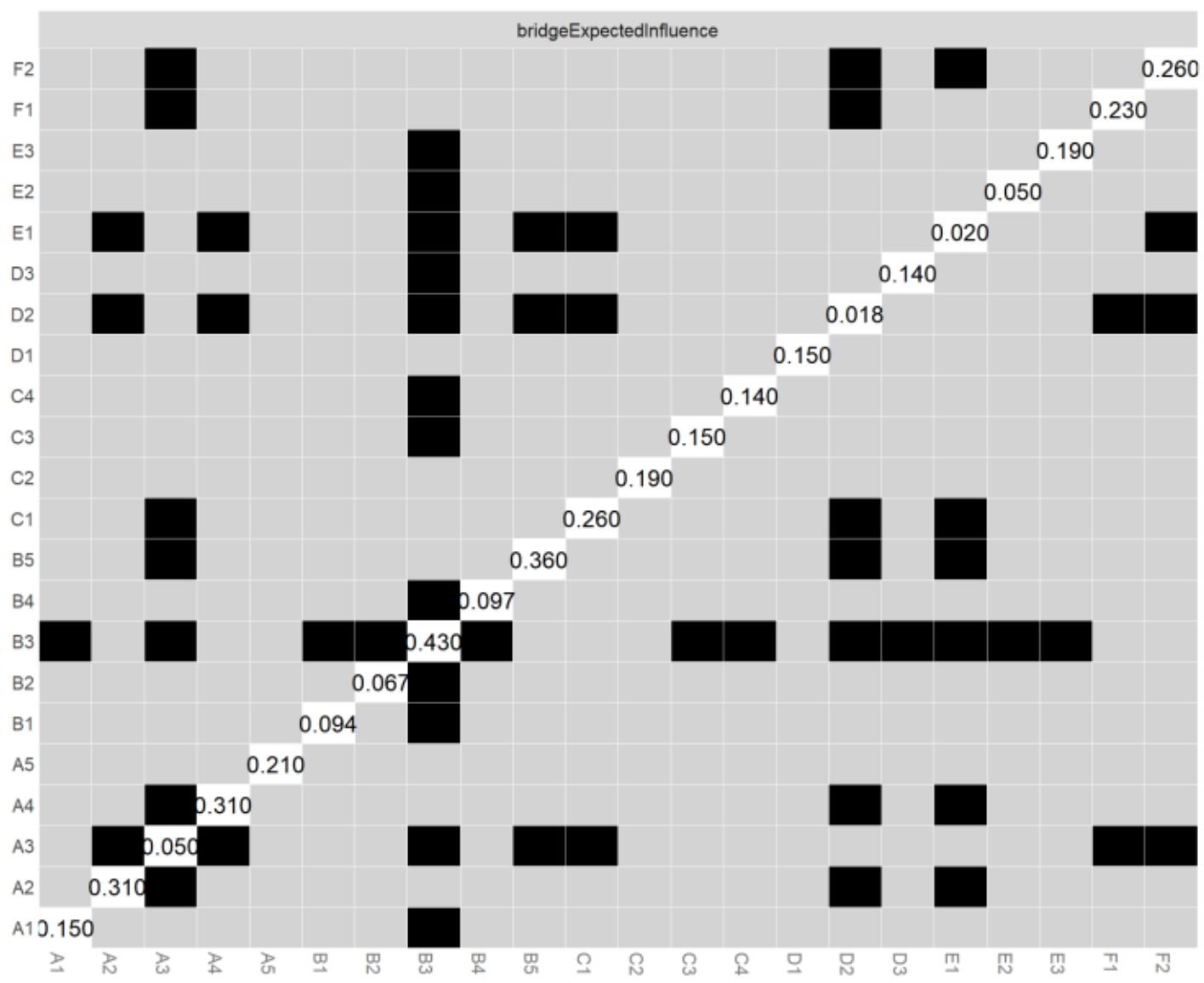

Abbreviations: A1, Feeling nervous; A2, Feeling irritable; A3, Feeling sad; A4, Difficulty sleeping;

A5, Worrying; B1, Difficulty concentrating; B2, Bradyphrenia; B3, Fatigue; B4, Feeling drowsy; B5, Pain; C1, Shortness of breath; C2, Chills; C3, Palpitations; C4, Sweating; D1, Skin changes; D2, "I don't look like myself"; D3, Hair loss; E1, Constipation; E2, Feeling bloated; E3, Lack of appetite; F1, Dizziness; F2, Noise sensitivity.

Figure S6e. Bootstrap difference test of edge weights

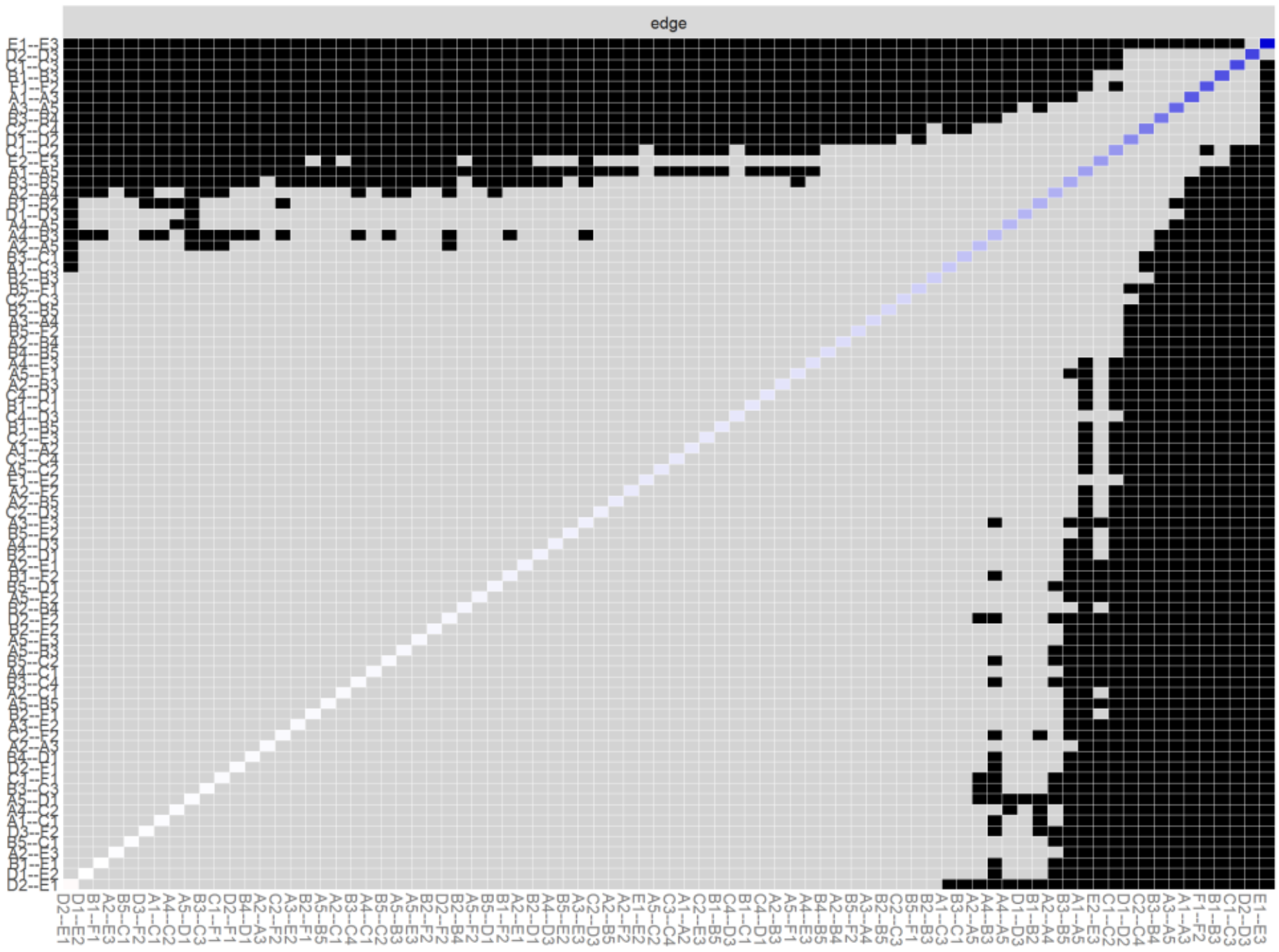

Abbreviations: A1, Feeling nervous; A2, Feeling irritable; A3, Feeling sad; A4, Difficulty sleeping; A5, Worrying; B1, Difficulty concentrating; B2, Bradyphrenia; B3, Fatigue; B4, Feeling drowsy; B5, Pain; C1, Shortness of breath; C2, Chills; C3, Palpitations; C4, Sweating; D1, Skin changes; D2, "I don't look like myself"; D3, Hair loss; E1, Constipation; E2, Feeling bloated; E3, Lack of appetite; F1, Dizziness; F2, Noise sensitivity.

Supplementary Material 7. Age- and sex-adjusted network sensitivity analysis

7.1. Network characteristics and pairwise comparisons

Table S7.1a. Characteristics of the three symptom networks

**Network**	**N**	**Nonzero edges**	**Negative edges**	**Global strength**
Original cor_auto	214	80	1	8.758
Unadjusted Pearson	214	60	0	6.733

Network	N	Nonzero edges	Negative edges	Global strength
Adjusted Pearson	214	41	0	5.251

Table S7.1b. Pairwise comparisons of network characteristics

Comparison	Edge r	Mean absolute change	Maximum absolute change	EI rho	Bridge EI rho
Reference vs adjusted (primary)	0.993	0.0065	0.0658	0.965	0.904
Original vs reference (method)	0.988	0.0104	0.1337	0.931	0.913
Original vs adjusted (combined)	0.978	0.0155	0.1952	0.905	0.820

7.2. Expected influence and rank comparisons

Table S7.2. Expected influence values and rankings across the three networks

Node	Symptom	Original value	Rank	Reference value	Rank	Adjusted value	Rank
A1	Feeling nervous	0.851	7	0.792	4	0.688	4
A2	Feeling irritable	0.736	14	0.588	10	0.431	13
A3	Feeling sad	0.949	5	0.802	3	0.701	3
A4	Difficulty sleeping	0.784	10	0.621	9	0.476	10
A5	Worrying	1.169	2	1.042	2	0.839	2
B1	Difficulty concentrating	0.774	12	0.671	8	0.530	7
B2	Bradyphrenia	0.511	21	0.391	20	0.280	20
B3	Fatigue	1.541	1	1.217	1	1.074	1
B4	Feeling drowsy	0.547	19	0.457	16	0.342	16
B5	Pain	0.823	8	0.711	7	0.516	8
C1	Shortness of breath	0.970	4	0.787	5	0.578	5
C2	Chills	0.872	6	0.582	11	0.417	15

Node	Symptom	Original value	Rank	Reference value	Rank	Adjusted value	Rank
C3	Palpitations	0.763	13	0.544	13	0.427	14
C4	Sweating	0.519	20	0.329	21	0.178	21
D1	Skin changes	0.624	18	0.420	19	0.331	17
D2	I don't look like myself	0.775	11	0.579	12	0.490	9
D3	Hair loss	0.791	9	0.532	14	0.441	11
E1	Constipation	0.703	15	0.531	15	0.439	12
E2	Feeling bloated	0.354	22	0.218	22	0.145	22
E3	Lack of appetite	1.072	3	0.771	6	0.574	6
F1	Dizziness	0.652	17	0.435	18	0.300	19
F2	Noise sensitivity	0.685	16	0.445	17	0.303	18

7.3. One-step bridge expected influence and rank comparisons

Table S7.3. One-step bridge expected influence values and rankings across the three networks

Node	Symptom	Original value	Rank	Reference value	Rank	Adjusted value	Rank
A1	Feeling nervous	0.148	12	0.125	8	0.084	6
A2	Feeling irritable	0.306	4	0.212	4	0.119	4
A3	Feeling sad	0.050	20	0.023	16	0.000	14
A4	Difficulty sleeping	0.309	3	0.195	5	0.110	5
A5	Worrying	0.206	8	0.172	6	0.035	11
B1	Difficulty concentrating	0.094	17	0.074	13	0.027	12
B2	Bradyphrenia	0.067	18	0.000	19	0.000	14

Node	Symptom	Original value	Rank	Reference value	Rank	Adjusted value	Rank
B3	Fatigue	0.430	1	0.303	1	0.244	1
B4	Feeling drowsy	0.097	16	0.080	12	0.039	10
B5	Pain	0.363	2	0.279	2	0.153	2
C1	Shortness of breath	0.260	5	0.254	3	0.120	3
C2	Chills	0.188	10	0.050	15	0.007	13
C3	Palpitations	0.148	11	0.091	11	0.063	7
C4	Sweating	0.138	15	0.067	14	0.000	14
D1	Skin changes	0.145	13	0.020	18	0.000	14
D2	I don't look like myself	0.018	22	0.000	19	0.000	14
D3	Hair loss	0.141	14	0.022	17	0.000	14
E1	Constipation	0.020	21	0.000	19	0.000	14
E2	Feeling bloated	0.050	19	0.000	19	0.000	14
E3	Lack of appetite	0.193	9	0.093	10	0.000	14
F1	Dizziness	0.225	7	0.121	9	0.052	9
F2	Noise sensitivity	0.259	6	0.130	7	0.054	8

7.4. Symptom pairs with the largest absolute changes in edge weights

Table S7.4. Ten symptom pairs with the largest absolute changes in edge weights after adjustment

Pair	Symptoms	Original	Reference	Adjusted	Absolute change
F1–F2	Dizziness / Noise sensitivity	0.4268	0.3144	0.2486	0.0658
E1–E3	Constipation / Lack of appetite	0.6289	0.4952	0.4337	0.0615

Pair	Symptoms	Original	Reference	Adjusted	Absolute change
C2–C4	Chills / Sweating	0.3244	0.2342	0.1775	0.0567
D1–D2	Skin changes / I don't look like myself	0.2926	0.2344	0.1901	0.0443
D2–D3	I don't look like myself / Hair loss	0.4642	0.3444	0.3001	0.0443
E2–E3	Feeling bloated / Lack of appetite	0.2503	0.1824	0.1405	0.0419
B1–B3	Difficulty concentrating / Fatigue	0.4271	0.3672	0.3260	0.0412
C1–C2	Shortness of breath / Chills	0.2566	0.2028	0.1630	0.0398
A4–E3	Difficulty sleeping / Lack of appetite	0.0758	0.0387	0.0000	0.0387
B1–B2	Difficulty concentrating / Bradyphrenia	0.1939	0.1424	0.1046	0.0379

Supplementary Material 8. Community assignments, redundancy screening, and node-removal sensitivity analyses

Table S8a. Node-to-community assignment

Node	Symptom	Community ID	PCA-derived symptom cluster
A1	Feeling nervous	1	Emotional-sleep
A2	Feeling irritable	1	Emotional-sleep
A3	Feeling sad	1	Emotional-sleep
A4	Difficulty sleeping	1	Emotional-sleep
A5	Worrying	1	Emotional-sleep
B1	Difficulty concentrating	2	Fatigue
B2	Bradyphrenia	2	Fatigue
B3	Fatigue	2	Fatigue
B4	Feeling drowsy	2	Fatigue
B5	Pain	2	Fatigue
C1	Shortness of breath	3	Physical
C2	Chills	3	Physical
C3	Palpitations	3	Physical
C4	Sweating	3	Physical
D1	Skin changes	4	Self-image
D2	I don't look like myself	4	Self-image
D3	Hair loss	4	Self-image
E1	Constipation	5	Gastrointestinal

Node	Symptom	Community ID	PCA-derived symptom cluster
E2	Feeling bloated	5	Gastrointestinal
E3	Lack of appetite	5	Gastrointestinal
F1	Dizziness	6	Neurological
F2	Noise sensitivity	6	Neurological

Table S8b. Screening of all 231 symptom pairs

**Node 1**	**Symptom 1**	**Node 2**	**Symptom 2**	**Pearson r**	**cor_auto r**	**Profile-difference proportion**	**Screening flag**	**Item-content considerations**
A3	Feeling sad	A1	Feeling nervous	0.705	0.752	0.200	Candidate	Nervousness and sadness describe different emotional experiences.
A4	Difficulty sleeping	A2	Feeling irritable	0.441	0.524	0.050	Candidate	Irritability describes emotional reactivity; difficulty sleeping describes a sleep-related experience.
A5	Worrying	A2	Feeling irritable	0.467	0.532	0.150	Candidate	Irritability and worrying describe emotional reactivity and worry-related cognition, respectively.
A5	Worrying	A4	Difficulty sleeping	0.511	0.573	0.100	Candidate	Difficulty sleeping and worrying describe sleep-related and cognitive experiences, respectively.
C3	Palpitations	C1	Shortness of breath	0.563	0.684	0.200	Candidate	Shortness of breath and palpitations describe respiratory discomfort and awareness of heartbeat, respectively.
D3	Hair loss	D1	Skin changes	0.454	0.502	0.150	Candidate	Both concern appearance, but skin changes and hair loss refer to different manifestations.

Node 1	Symptom 1	Node 2	Symptom 2	Pearson r	cor_auto r	Profile-difference proportion	Screening flag	Item-content considerations
D3	Hair loss	D2	I don't look like myself	0.583	0.667	0.150	Candidate	Perceived overall change in appearance may partly overlap with hair loss; the latter is a specific manifestation. Retention was examined through node-removal analyses.
F2	Noise sensitivity	F1	Dizziness	0.511	0.582	0.000	Candidate	Dizziness and noise sensitivity describe different symptom experiences.
B4	Feeling drowsy	B3	Fatigue	0.530	0.643	0.600	Not flagged	Fatigue describes exhaustion or lack of energy; feeling drowsy describes sleepiness. This pair was not flagged; a model excluding feeling drowsy additionally examined its influence.
A2	Feeling irritable	A1	Feeling nervous	0.381	0.432	0.300	Not flagged	
A4	Difficulty sleeping	A1	Feeling nervous	0.338	0.396	0.200	Not flagged	
A5	Worrying	A1	Feeling nervous	0.636	0.696	0.350	Not flagged	
B1	Difficulty concentrating	A1	Feeling nervous	0.140	0.160	0.350	Not flagged	
B2	Bradyphrenia	A1	Feeling nervous	0.077	0.089	0.450	Not flagged	
B3	Fatigue	A1	Feeling nervous	0.254	0.340	0.400	Not flagged	
B4	Feeling drowsy	A1	Feeling nervous	0.144	0.159	0.350	Not flagged	

Node 1	Symptom 1	Node 2	Symptom 2	Pearson r	cor_auto r	Profile-difference proportion	Screening flag	Item-content considerations
B5	Pain	A1	Feeling nervous	0.194	0.213	0.450	Not flagged	
C1	Shortness of breath	A1	Feeling nervous	0.300	0.346	0.400	Not flagged	
C2	Chills	A1	Feeling nervous	0.197	0.228	0.250	Not flagged	
C3	Palpitations	A1	Feeling nervous	0.332	0.403	0.300	Not flagged	
C4	Sweating	A1	Feeling nervous	0.071	0.070	0.250	Not flagged	
D1	Skin changes	A1	Feeling nervous	0.111	0.126	0.450	Not flagged	
D2	I don't look like myself	A1	Feeling nervous	0.050	0.047	0.550	Not flagged	
D3	Hair loss	A1	Feeling nervous	0.088	0.101	0.600	Not flagged	
E1	Constipation	A1	Feeling nervous	0.123	0.147	0.500	Not flagged	
E2	Feeling bloated	A1	Feeling nervous	0.113	0.132	0.450	Not flagged	
E3	Lack of appetite	A1	Feeling nervous	0.177	0.199	0.300	Not flagged	
F1	Dizziness	A1	Feeling nervous	0.160	0.184	0.350	Not flagged	
F2	Noise sensitivity	A1	Feeling nervous	0.143	0.159	0.300	Not flagged	
A3	Feeling sad	A2	Feeling irritable	0.378	0.444	0.250	Not flagged	
B1	Difficulty concentrating	A2	Feeling irritable	0.255	0.298	0.300	Not flagged	
B2	Bradyphrenia	A2	Feeling irritable	0.134	0.147	0.300	Not flagged	
B3	Fatigue	A2	Feeling irritable	0.353	0.454	0.350	Not flagged	
B4	Feeling drowsy	A2	Feeling irritable	0.322	0.375	0.400	Not flagged	

Node 1	Symptom 1	Node 2	Symptom 2	Pearson r	cor_auto r	Profile-difference proportion	Screening flag	Item-content considerations
B5	Pain	A2	Feeling irritable	0.311	0.356	0.450	Not flagged	
C1	Shortness of breath	A2	Feeling irritable	0.264	0.313	0.250	Not flagged	
C2	Chills	A2	Feeling irritable	0.176	0.214	0.450	Not flagged	
C3	Palpitations	A2	Feeling irritable	0.116	0.153	0.250	Not flagged	
C4	Sweating	A2	Feeling irritable	0.102	0.107	0.500	Not flagged	
D1	Skin changes	A2	Feeling irritable	0.105	0.127	0.600	Not flagged	
D2	I don't look like myself	A2	Feeling irritable	0.030	0.025	0.650	Not flagged	
D3	Hair loss	A2	Feeling irritable	0.042	0.075	0.650	Not flagged	
E1	Constipation	A2	Feeling irritable	0.180	0.238	0.650	Not flagged	
E2	Feeling bloated	A2	Feeling irritable	0.089	0.113	0.600	Not flagged	
E3	Lack of appetite	A2	Feeling irritable	0.210	0.259	0.500	Not flagged	
F1	Dizziness	A2	Feeling irritable	0.178	0.203	0.400	Not flagged	
F2	Noise sensitivity	A2	Feeling irritable	0.231	0.274	0.400	Not flagged	
A4	Difficulty sleeping	A3	Feeling sad	0.431	0.509	0.250	Not flagged	
A5	Worrying	A3	Feeling sad	0.676	0.763	0.300	Not flagged	
B1	Difficulty concentrating	A3	Feeling sad	0.165	0.186	0.350	Not flagged	
B2	Bradyphrenia	A3	Feeling sad	0.074	0.077	0.350	Not flagged	
B3	Fatigue	A3	Feeling sad	0.245	0.337	0.400	Not flagged	
B4	Feeling drowsy	A3	Feeling sad	0.153	0.182	0.400	Not flagged	
B5	Pain	A3	Feeling sad	0.153	0.170	0.500	Not flagged	

Node 1	Symptom 1	Node 2	Symptom 2	Pearson r	cor_auto r	Profile-difference proportion	Screening flag	Item-content considerations
C1	Shortness of breath	A3	Feeling sad	0.235	0.267	0.500	Not flagged	
C2	Chills	A3	Feeling sad	0.145	0.172	0.350	Not flagged	
C3	Palpitations	A3	Feeling sad	0.244	0.301	0.400	Not flagged	
C4	Sweating	A3	Feeling sad	0.056	0.065	0.300	Not flagged	
D1	Skin changes	A3	Feeling sad	0.086	0.103	0.400	Not flagged	
D2	I don't look like myself	A3	Feeling sad	0.034	0.020	0.550	Not flagged	
D3	Hair loss	A3	Feeling sad	0.070	0.097	0.600	Not flagged	
E1	Constipation	A3	Feeling sad	0.124	0.148	0.550	Not flagged	
E2	Feeling bloated	A3	Feeling sad	0.161	0.201	0.450	Not flagged	
E3	Lack of appetite	A3	Feeling sad	0.248	0.292	0.350	Not flagged	
F1	Dizziness	A3	Feeling sad	0.156	0.192	0.450	Not flagged	
F2	Noise sensitivity	A3	Feeling sad	0.148	0.172	0.350	Not flagged	
B1	Difficulty concentrating	A4	Difficulty sleeping	0.286	0.330	0.400	Not flagged	
B2	Bradyphrenia	A4	Difficulty sleeping	0.143	0.156	0.400	Not flagged	
B3	Fatigue	A4	Difficulty sleeping	0.389	0.515	0.400	Not flagged	
B4	Feeling drowsy	A4	Difficulty sleeping	0.267	0.300	0.400	Not flagged	
B5	Pain	A4	Difficulty sleeping	0.292	0.338	0.400	Not flagged	
C1	Shortness of breath	A4	Difficulty sleeping	0.271	0.341	0.300	Not flagged	
C2	Chills	A4	Difficulty sleeping	0.226	0.283	0.400	Not flagged	
C3	Palpitations	A4	Difficulty sleeping	0.218	0.289	0.450	Not flagged	
C4	Sweating	A4	Difficulty sleeping	0.076	0.075	0.450	Not flagged	

Node 1	Symptom 1	Node 2	Symptom 2	Pearson r	cor_auto r	Profile-difference proportion	Screening flag	Item-content considerations
D1	Skin changes	A4	Difficulty sleeping	0.081	0.094	0.600	Not flagged	
D2	I don't look like myself	A4	Difficulty sleeping	0.076	0.060	0.750	Not flagged	
D3	Hair loss	A4	Difficulty sleeping	0.136	0.192	0.750	Not flagged	
E1	Constipation	A4	Difficulty sleeping	0.208	0.247	0.800	Not flagged	
E2	Feeling bloated	A4	Difficulty sleeping	0.141	0.175	0.650	Not flagged	
E3	Lack of appetite	A4	Difficulty sleeping	0.254	0.317	0.600	Not flagged	
F1	Dizziness	A4	Difficulty sleeping	0.169	0.214	0.400	Not flagged	
F2	Noise sensitivity	A4	Difficulty sleeping	0.190	0.228	0.500	Not flagged	
B1	Difficulty concentrating	A5	Worrying	0.219	0.250	0.400	Not flagged	
B2	Bradyphrenia	A5	Worrying	0.058	0.062	0.300	Not flagged	
B3	Fatigue	A5	Worrying	0.302	0.411	0.400	Not flagged	
B4	Feeling drowsy	A5	Worrying	0.259	0.283	0.400	Not flagged	
B5	Pain	A5	Worrying	0.289	0.324	0.450	Not flagged	
C1	Shortness of breath	A5	Worrying	0.249	0.298	0.450	Not flagged	
C2	Chills	A5	Worrying	0.266	0.323	0.350	Not flagged	
C3	Palpitations	A5	Worrying	0.241	0.287	0.450	Not flagged	
C4	Sweating	A5	Worrying	0.035	0.025	0.400	Not flagged	
D1	Skin changes	A5	Worrying	0.137	0.164	0.650	Not flagged	
D2	I don't look like myself	A5	Worrying	0.081	0.087	0.750	Not flagged	
D3	Hair loss	A5	Worrying	0.076	0.117	0.700	Not flagged	
E1	Constipation	A5	Worrying	0.131	0.154	0.750	Not flagged	

Node 1	Symptom 1	Node 2	Symptom 2	Pearson r	cor_auto r	Profile-difference proportion	Screening flag	Item-content considerations
E2	Feeling bloated	A5	Worrying	0.132	0.150	0.650	Not flagged	
E3	Lack of appetite	A5	Worrying	0.254	0.293	0.650	Not flagged	
F1	Dizziness	A5	Worrying	0.257	0.302	0.400	Not flagged	
F2	Noise sensitivity	A5	Worrying	0.252	0.279	0.350	Not flagged	
B2	Bradyphrenia	B1	Difficulty concentrating	0.440	0.504	0.300	Not flagged	
B3	Fatigue	B1	Difficulty concentrating	0.653	0.740	0.450	Not flagged	
B4	Feeling drowsy	B1	Difficulty concentrating	0.351	0.408	0.150	Not flagged	
B5	Pain	B1	Difficulty concentrating	0.418	0.477	0.100	Not flagged	
C1	Shortness of breath	B1	Difficulty concentrating	0.340	0.415	0.350	Not flagged	
C2	Chills	B1	Difficulty concentrating	0.233	0.268	0.300	Not flagged	
C3	Palpitations	B1	Difficulty concentrating	0.174	0.233	0.350	Not flagged	
C4	Sweating	B1	Difficulty concentrating	0.180	0.182	0.450	Not flagged	
D1	Skin changes	B1	Difficulty concentrating	0.103	0.119	0.550	Not flagged	
D2	I don't look like myself	B1	Difficulty concentrating	0.061	0.051	0.600	Not flagged	
D3	Hair loss	B1	Difficulty concentrating	0.064	0.083	0.500	Not flagged	
E1	Constipation	B1	Difficulty concentrating	0.121	0.125	0.450	Not flagged	
E2	Feeling bloated	B1	Difficulty concentrating	0.030	0.033	0.500	Not flagged	
E3	Lack of appetite	B1	Difficulty concentrating	0.123	0.129	0.350	Not flagged	
F1	Dizziness	B1	Difficulty concentrating	0.207	0.251	0.300	Not flagged	

Node 1	Symptom 1	Node 2	Symptom 2	Pearson r	cor_auto r	Profile-difference proportion	Screening flag	Item-content considerations
F2	Noise sensitivity	B1	Difficulty concentrating	0.227	0.269	0.250	Not flagged	
B3	Fatigue	B2	Bradyphrenia	0.453	0.503	0.600	Not flagged	
B4	Feeling drowsy	B2	Bradyphrenia	0.308	0.349	0.150	Not flagged	
B5	Pain	B2	Bradyphrenia	0.367	0.404	0.200	Not flagged	
C1	Shortness of breath	B2	Bradyphrenia	0.223	0.271	0.350	Not flagged	
C2	Chills	B2	Bradyphrenia	0.076	0.083	0.300	Not flagged	
C3	Palpitations	B2	Bradyphrenia	0.155	0.202	0.400	Not flagged	
C4	Sweating	B2	Bradyphrenia	0.102	0.097	0.250	Not flagged	
D1	Skin changes	B2	Bradyphrenia	0.150	0.181	0.350	Not flagged	
D2	I don't look like myself	B2	Bradyphrenia	0.108	0.131	0.450	Not flagged	
D3	Hair loss	B2	Bradyphrenia	0.045	0.065	0.400	Not flagged	
E1	Constipation	B2	Bradyphrenia	0.033	0.032	0.450	Not flagged	
E2	Feeling bloated	B2	Bradyphrenia	0.072	0.091	0.400	Not flagged	
E3	Lack of appetite	B2	Bradyphrenia	0.112	0.136	0.450	Not flagged	
F1	Dizziness	B2	Bradyphrenia	0.200	0.226	0.200	Not flagged	
F2	Noise sensitivity	B2	Bradyphrenia	0.195	0.234	0.250	Not flagged	
B5	Pain	B3	Fatigue	0.491	0.590	0.350	Not flagged	
C1	Shortness of breath	B3	Fatigue	0.377	0.502	0.400	Not flagged	
C2	Chills	B3	Fatigue	0.220	0.295	0.400	Not flagged	
C3	Palpitations	B3	Fatigue	0.242	0.372	0.400	Not flagged	
C4	Sweating	B3	Fatigue	0.232	0.233	0.700	Not flagged	
D1	Skin changes	B3	Fatigue	0.114	0.169	0.800	Not flagged	
D2	I don't look like myself	B3	Fatigue	0.054	0.073	0.800	Not flagged	
D3	Hair loss	B3	Fatigue	0.039	0.077	0.750	Not flagged	

Node 1	Symptom 1	Node 2	Symptom 2	Pearson r	cor_auto r	Profile-difference proportion	Screening flag	Item-content considerations
E1	Constipation	B3	Fatigue	0.129	0.136	0.850	Not flagged	
E2	Feeling bloated	B3	Fatigue	0.081	0.121	0.750	Not flagged	
E3	Lack of appetite	B3	Fatigue	0.177	0.216	0.650	Not flagged	
F1	Dizziness	B3	Fatigue	0.188	0.275	0.550	Not flagged	
F2	Noise sensitivity	B3	Fatigue	0.216	0.285	0.550	Not flagged	
B5	Pain	B4	Feeling drowsy	0.388	0.440	0.100	Not flagged	
C1	Shortness of breath	B4	Feeling drowsy	0.216	0.268	0.200	Not flagged	
C2	Chills	B4	Feeling drowsy	0.171	0.195	0.450	Not flagged	
C3	Palpitations	B4	Feeling drowsy	0.168	0.221	0.350	Not flagged	
C4	Sweating	B4	Feeling drowsy	0.167	0.180	0.450	Not flagged	
D1	Skin changes	B4	Feeling drowsy	0.143	0.164	0.400	Not flagged	
D2	I don't look like myself	B4	Feeling drowsy	0.035	0.021	0.550	Not flagged	
D3	Hair loss	B4	Feeling drowsy	0.034	0.043	0.450	Not flagged	
E1	Constipation	B4	Feeling drowsy	0.006	0.018	0.350	Not flagged	
E2	Feeling bloated	B4	Feeling drowsy	0.091	0.101	0.350	Not flagged	
E3	Lack of appetite	B4	Feeling drowsy	0.081	0.092	0.300	Not flagged	
F1	Dizziness	B4	Feeling drowsy	0.166	0.191	0.150	Not flagged	
F2	Noise sensitivity	B4	Feeling drowsy	0.116	0.129	0.100	Not flagged	
C1	Shortness of breath	B5	Pain	0.279	0.330	0.300	Not flagged	
C2	Chills	B5	Pain	0.219	0.254	0.450	Not flagged	

Node 1	Symptom 1	Node 2	Symptom 2	Pearson r	cor_auto r	Profile-difference proportion	Screening flag	Item-content considerations
C3	Palpitations	B5	Pain	0.162	0.224	0.550	Not flagged	
C4	Sweating	B5	Pain	0.135	0.124	0.500	Not flagged	
D1	Skin changes	B5	Pain	0.156	0.185	0.600	Not flagged	
D2	I don't look like myself	B5	Pain	0.138	0.146	0.750	Not flagged	
D3	Hair loss	B5	Pain	0.062	0.083	0.650	Not flagged	
E1	Constipatio n	B5	Pain	0.119	0.137	0.550	Not flagged	
E2	Feeling bloated	B5	Pain	0.151	0.184	0.550	Not flagged	
E3	Lack of appetite	B5	Pain	0.099	0.105	0.450	Not flagged	
F1	Dizziness	B5	Pain	0.313	0.364	0.400	Not flagged	
F2	Noise sensitivity	B5	Pain	0.311	0.354	0.350	Not flagged	
C2	Chills	C1	Shortness of breath	0.456	0.549	0.300	Not flagged	
C4	Sweating	C1	Shortness of breath	0.221	0.240	0.600	Not flagged	
D1	Skin changes	C1	Shortness of breath	0.121	0.154	0.450	Not flagged	
D2	I don't look like myself	C1	Shortness of breath	-0.006	-0.014	0.750	Not flagged	
D3	Hair loss	C1	Shortness of breath	0.042	0.051	0.650	Not flagged	
E1	Constipatio n	C1	Shortness of breath	0.099	0.123	0.650	Not flagged	
E2	Feeling bloated	C1	Shortness of breath	0.078	0.096	0.500	Not flagged	
E3	Lack of appetite	C1	Shortness of breath	0.165	0.191	0.450	Not flagged	
F1	Dizziness	C1	Shortness of breath	0.188	0.215	0.350	Not flagged	
F2	Noise sensitivity	C1	Shortness of breath	0.149	0.181	0.300	Not flagged	
C3	Palpitations	C2	Chills	0.383	0.470	0.200	Not flagged	
C4	Sweating	C2	Chills	0.435	0.504	0.400	Not flagged	

Node 1	Symptom 1	Node 2	Symptom 2	Pearson r	cor_auto r	Profile-difference proportion	Screening flag	Item-content considerations
D1	Skin changes	C2	Chills	0.070	0.071	0.350	Not flagged	
D2	I don't look like myself	C2	Chills	0.089	0.097	0.650	Not flagged	
D3	Hair loss	C2	Chills	0.179	0.224	0.550	Not flagged	
E1	Constipation	C2	Chills	0.153	0.189	0.550	Not flagged	
E2	Feeling bloated	C2	Chills	0.091	0.105	0.400	Not flagged	
E3	Lack of appetite	C2	Chills	0.198	0.251	0.350	Not flagged	
F1	Dizziness	C2	Chills	0.141	0.175	0.250	Not flagged	
F2	Noise sensitivity	C2	Chills	0.158	0.194	0.250	Not flagged	
C4	Sweating	C3	Palpitations	0.264	0.314	0.300	Not flagged	
D1	Skin changes	C3	Palpitations	0.078	0.113	0.400	Not flagged	
D2	I don't look like myself	C3	Palpitations	0.015	0.019	0.650	Not flagged	
D3	Hair loss	C3	Palpitations	-0.014	0.009	0.350	Not flagged	
E1	Constipation	C3	Palpitations	0.060	0.092	0.450	Not flagged	
E2	Feeling bloated	C3	Palpitations	0.087	0.117	0.400	Not flagged	
E3	Lack of appetite	C3	Palpitations	0.084	0.119	0.300	Not flagged	
F1	Dizziness	C3	Palpitations	0.107	0.142	0.250	Not flagged	
F2	Noise sensitivity	C3	Palpitations	0.122	0.159	0.250	Not flagged	
D1	Skin changes	C4	Sweating	0.212	0.235	0.200	Not flagged	
D2	I don't look like myself	C4	Sweating	0.082	0.061	0.250	Not flagged	
D3	Hair loss	C4	Sweating	0.214	0.247	0.250	Not flagged	
E1	Constipation	C4	Sweating	0.022	0.006	0.250	Not flagged	
E2	Feeling bloated	C4	Sweating	0.079	0.107	0.250	Not flagged	

Node 1	Symptom 1	Node 2	Symptom 2	Pearson r	cor_auto r	Profile-difference proportion	Screening flag	Item-content considerations
E3	Lack of appetite	C4	Sweating	0.027	0.027	0.400	Not flagged	
F1	Dizziness	C4	Sweating	0.111	0.132	0.250	Not flagged	
F2	Noise sensitivity	C4	Sweating	0.096	0.103	0.200	Not flagged	
D2	I don't look like myself	D1	Skin changes	0.498	0.552	0.300	Not flagged	
E1	Constipation	D1	Skin changes	0.073	0.085	0.250	Not flagged	
E2	Feeling bloated	D1	Skin changes	0.089	0.117	0.200	Not flagged	
E3	Lack of appetite	D1	Skin changes	0.050	0.063	0.350	Not flagged	
F1	Dizziness	D1	Skin changes	0.062	0.075	0.200	Not flagged	
F2	Noise sensitivity	D1	Skin changes	0.043	0.060	0.150	Not flagged	
E1	Constipation	D2	I don't look like myself	-0.084	-0.112	0.300	Not flagged	
E2	Feeling bloated	D2	I don't look like myself	0.036	0.034	0.200	Not flagged	
E3	Lack of appetite	D2	I don't look like myself	-0.074	-0.091	0.450	Not flagged	
F1	Dizziness	D2	I don't look like myself	0.130	0.154	0.500	Not flagged	
F2	Noise sensitivity	D2	I don't look like myself	0.129	0.165	0.500	Not flagged	
E1	Constipation	D3	Hair loss	-0.076	-0.087	0.300	Not flagged	
E2	Feeling bloated	D3	Hair loss	0.011	0.024	0.200	Not flagged	
E3	Lack of appetite	D3	Hair loss	-0.019	0.008	0.300	Not flagged	
F1	Dizziness	D3	Hair loss	0.116	0.146	0.300	Not flagged	
F2	Noise sensitivity	D3	Hair loss	0.105	0.159	0.350	Not flagged	
E2	Feeling bloated	E1	Constipation	0.332	0.404	0.050	Not flagged	

Node 1	Symptom 1	Node 2	Symptom 2	Pearson r	cor_auto r	Profile-difference proportion	Screening flag	Item-content considerations
E3	Lack of appetite	E1	Constipation	0.695	0.786	0.350	Not flagged	
F1	Dizziness	E1	Constipation	-0.043	-0.056	0.350	Not flagged	
F2	Noise sensitivity	E1	Constipation	-0.003	-0.006	0.350	Not flagged	
E3	Lack of appetite	E2	Feeling bloated	0.415	0.490	0.250	Not flagged	
F1	Dizziness	E2	Feeling bloated	0.021	0.027	0.250	Not flagged	
F2	Noise sensitivity	E2	Feeling bloated	0.027	0.031	0.200	Not flagged	
F1	Dizziness	E3	Lack of appetite	0.070	0.095	0.250	Not flagged	
F2	Noise sensitivity	E3	Lack of appetite	0.105	0.119	0.250	Not flagged	

Table S8c. Single-node-removal sensitivity analyses

Removed node	Removed symptom	EI rank rho	BEI rank rho	Fatigue EI rank	Fatigue BEI rank	Singleton community	Reason for omission
A1	Feeling nervous	0.988	0.956	1	1	No	Node in a flagged pair
A2	Feeling irritable	0.987	0.968	1	1	No	Node in a flagged pair
A3	Feeling sad	0.958	0.996	1	1	No	Node in a flagged pair
A4	Difficulty sleeping	0.991	0.978	1	3	No	Node in a flagged pair
A5	Worrying	0.997	0.988	1	1	No	Node in a flagged pair
C1	Shortness of breath	0.978	0.962	1	1	No	Node in a flagged pair
C3	Palpitations	0.938	0.965	1	1	No	Node in a flagged pair
D1	Skin changes	0.956	0.987	1	1	No	Node in a flagged pair
D2	I don't look like myself	0.952	0.934	1	1	No	Node in a flagged pair
D3	Hair loss	0.914	0.962	1	1	No	Node in a flagged pair
F1	Dizziness	0.984	0.970	1	1	Yes	Node in a flagged pair
F2	Noise sensitivity	0.986	0.984	1	1	Yes	Node in a flagged pair
B4	Feeling drowsy	0.986	0.997	1	1	No	Fatigue–drowsiness assessment
